# Antitumor evaluation of two selected Pakistani plant extracts on human bone and breast cancer cell lines

**DOI:** 10.1186/s12906-016-1215-9

**Published:** 2016-07-26

**Authors:** Nadja Engel, Iftikhar Ali, Anna Adamus, Marcus Frank, Akber Dad, Sajjad Ali, Barbara Nebe, Muhammad Atif, Muhammad Ismail, Peter Langer, Viqar Uddin Ahmad

**Affiliations:** 1 Department of Pediatric Surgery, University Hospital Marburg, Baldingerstraße, Marburg, 35034 Germany; 2 Department of Chemistry, Karakoram International University, Gilgit-Baltistan, 15100 Pakistan; 3 Institut für Chemie, Universität Rostock, Albert-Einstein-Str. 3a, Rostock, 18059 Germany; 4 Department of Cell Biology, University Medical Center Rostock, Schillingallee 69, Rostock, 18057 Germany; 5 Medical Biology and Electron Microscopy Centre, University Medical Center Rostock, Strempelstraße 14, Rostock, 18057 Germany; 6 University of Education Vehari Campus, Punjab, Pakistan; 7 HEJ Research Institute of Chemistry, ICCBS, University of Karachi, Karachi, 75270 Pakistan

**Keywords:** Traditional herbal remedies, Plant extracts, Antitumor evaluation, Actin cytoskeleton

## Abstract

**Background:**

The medicinal plants *Vincetoxicum arnottianum* (VSM), *Berberis orthobotrys* (BORM), *Onosma hispida* (OHRM and OHAM) and *Caccinia macranthera* (CMM) are used traditionally in Pakistan and around the world for the treatment of various diseases including cancer, dermal infections, uterine tumor, wounds etc. The present study focuses on the investigation of the selected Pakistani plants for their potential as anticancer agents on human bone and breast cancer cell lines in comparison with non-tumorigenic control cells.

**Methods:**

The antitumor evaluation was carried out on human bone (MG-63, Saos-2) and breast cancer cell lines (MCF-7, BT-20) in contrast to non-tumorigenic control cells (POB, MCF-12A) via cell viability measurements, cell cycle analysis, Annexin V/PI staining, microscopy based methods as well as migration/invasion determination, metabolic live cell monitoring and western blotting.

**Results:**

After the first initial screening of the plant extracts, two extracts (BORM, VSM) revealed the highest potential with regard to its antitumor activity. Both extracts caused a significant reduction of cell viability in the breast and bone cancer cells in a concentration dependent manner. The effect of VSM is achieved primarily by inducing a G2/M arrest in the cell cycle and the stabilization of the actin stress fibers leading to reduced cell motility. By contrast BORM’s cytotoxic properties were caused through the lysosomal-mediated cell death pathway indicated by an upregulation of Bcl-2 expression.

**Conclusions:**

The antitumor evaluation of certain medicinal plants presented in this study identified the methanolic root extract of *Berberis orthobotrys* and the methanolic extract of *Vincetoxicum arnottianum* as promising sources for exhibiting the antitumor activity. Therefore, the indigenous use of the herbal remedies for the treatment of cancer and cancer-related diseases has a scientific basis. Moreover, the present study provides a base for phytochemical investigation of the plant extracts.

**Electronic supplementary material:**

The online version of this article (doi:10.1186/s12906-016-1215-9) contains supplementary material, which is available to authorized users.

## Background

Natural products have historically and continually been investigated for promising new leads in pharmaceutical development. Cancer is a major public health problem worldwide with millions of new cancer patients diagnosed each year and many deaths resulting from this disease. Chemotherapy remains the principal mode of treatment for various cancers. Researchers have focused on the anticancer activity of the plants because the medicinal plants are used in different countries for the treatment and prevention of cancer [1, 2]. For example, Traditional Chinese medicine (TCM) is used as an adjuvant therapy to alleviate cancer symptoms at the terminal stages when Western medicine treatments cannot offer any other treatment options [3, 4].

In Pakistan, as in other developing countries, traditional medicines are in widespread use; with the practitioners formulating and dispensing the recipes to their patients. The medicaments are prepared most often from a combination of two or more plant products which may contain active chemical constituents with multiple physiological and pharmacological activities and could be used in treating various disease conditions. The discovery of effective herbs and elucidation of their underlying mechanisms could lead to the development of an alternative and complementary method for cancer prevention and/or treatment. Based on an analysis of published literature, we selected four traditional Pakistani plants with medicinal value to evaluate their anticancer efficacy. In search of the target plant extracts for the development of anticancer drugs, here we have investigated *Vincetoxicum arnottianum*, *Berberis orthobotrys*, *Onosma hispida* and *Caccinia macranthera* of Pakistan origin.

*Vincetoxicum arnottianum* Wight (Syn: *Cyanchum arnottianum* Wight) is a perennial plant of the Apocynaceae family found in different parts of Pakistan including Hazara, Swat, Kaghan, Shinkiari, Kashmir etc. [5]. The family Apocynaceae is one of the largest angiosperm family comprising 375 genera and over 5100 species. Plants of the family Apocynaceae have been reported to be extensively used for the treatment of the skin diseases, pimples [6], malaria, diabetes and diarrhea and most importantly some species have been used in cancer chemotherapy [7]. Some species of *Vincetoxicum* have exhibited very high cytotoxicity against brine shrimps [16], antidiarrheal and antispasmodic [8], antibiotic [9], anti-inflammatory [10], antidiabetic and antioxidant [11] activities etc. Alkaloids are normally reported from various *Vincetoxicum* species [12, 13]. The plant *V. arnottianum* (syn. *C. arnottianum*) has been reported for the treatment of maggots in wounds of cattle, horses and sheep [14], wounds and injuries [15] etc.

*Berberis orthobotrys* Bien ex Aitch. is a shrub that belongs to the family Berberidaceae. Berberidaceae family comprises 13 genera and 650 species [25] and it is represented in Pakistan by 3 genera and 22 species. Various species of the genus *Berberis* are reported from different parts of Pakistan i.e. Gilgit, Baltistan, Chitral, Skardu, Astor etc. Hussain et al. [16] have studied the diversity and ecological characteristics of different plants including *B. orthobotrys*. Mokhber-Dezfuli et al. [17] and Srivastava et al. [18] have reviewed on the chemical and biological diversity in *Berberis*. The plant *B. orthobotrys* has been reported for the treatment of ulcer, stomach problems, kidney stones, uterine tumor, wounds [19], blood purification, jaundice, urine problem, diarrhea [20], gastrointestinal diseases [21] etc. Moreover the plant *B. orthobotrys* has revealed various biological activities including antihypertensive [22], cardiac depressant [23], antihyperlipidemic [24] etc. The chemical constituents that are reported from *B. orthobotrys* include alkaloids [25].

*Onosma hispida* Wall. ex G. Don. is a perennial herb of the Boraginaceae family found in different localities in Pakistan including Gilgit, Chitral, Baluchistan, Swat, Hazara etc. Kumar et al. [26] have reviewed the genus for its phytochemical and pharmacological aspects. The genus *Onosma* L. is one of the largest and most species-rich genera of the family Boraginaceae comprising more than 150 species [27–29]. *O. hispida* is used as a medicinal herb [30, 31] exhibiting various biological properties including antibacterial activity [32]. The plant *O. hispida* has been reported to be used as blood purifier and for cuts, swells, wounds [33]. And it has also been reported for the treatment of abdominal ulcers, hair problems, bladder and kidney stones and rheumatism [34], pneumonia, typhoid fever and also used for dyeing hairs [35]. A number of chemical constituents including benzoic acid derivatives, apigenin derivatives, flavones and flavanone derivatives have been isolated from *O. hispida* [26].

*Caccinia macranthera* (Banks & Sol.) Brand (Syn: *Borago macranthera* Banks & Sol.) is a leafy perennial plant of the Boraginaceae family found in Baluchistan province in Pakistan [36]. The roots of *C. macranthera* have been reported to be used for the treatment of dermal infections, liver disorders and dyspepsia and some other traditional uses [37, 38], sedative, treatment of cough, expectorant [39]. Moreover, the leaves of *C. macranthera* have also been reported for its medicinal properties [40]. The Boraginaceae is a large family that comprises approximately 205 genera and 2500 species worldwide [41]. The root extract of *C. macranthera* was studied for induction of phage production [42]. Different chemical constituents including glycosides [43], pyrrolizidine alkaloids [44], triterpenoid sapogenin [45] have been reported from the species of the genus *Caccinia* other than *C. macranthera*. However El-Shazly & Wink have reported that pyrrolizidine alkaloids are commonly found in Boraginaceae family. However, the overview about the medicinal plants *Vincetoxicum arnottianum*, *Berberis orthobotrys*, *Onosma hispida* and *Caccinia macranthera* of Pakistan origin is given in Table 1.

**Table 1:** Overview of the selected Pakistani plants used in this study

Plant name	Sample code	Description	Family	Medicinal uses
*Vincetoxicum arnottianum* Wight	VSM	Methanolic extract of the plant.	Apocynaceae	Wounds, Injuries, Maggots in wounds of cattle, horses etc.
*Berberis orthobotrys* Bien. ex Aitch.	BORM	Methanolic root extract of the plant.	Berberidaceae	Uterine tumor, wounds, gastrointestinal problems, ulcer, blood purification, jaundice, urine problem, diarrhea, antihypertensive, cardiac depressant, antihyperlipidemic etc
	BOFM	Methanolic extract of the flowers of the plant		
	BO-5	Ethylacetate soluble oily substance extracted from the methanolic fruit extract of the plant.		
	BO-23	n-hexane soluble oily substance extracted from the methanolic fruit extract of the plant.		
*Onosma hispida* Wall. ex G. Don.	OHRM	Methanolic root extract of the plant.	Berberidaceae	Wounds, cuts, swells, abdominal ulcer, antibacterial, blood purifier, hair problems, dying hair, bladder and kidney stones, rheumatism, pneumonia, typhoid fever etc.
	OHAM	Methanolic extract of the aerial parts of the plant.		
*Caccinia macranthera* (Banks & Sol.) Brand	CMM	Methanolic extract of the plant.	Boraginaceae	Dermal infections, liver disorders, dyspepsia, sedative, cough, expectorant, induction of phage production etc

Despite their widespread use, however, no scientific assessment for anticancer effect has been conducted in most cases. Considering their increasing recognition and consumption, the present study was undertaken to evaluate the anticancer potential of these plant extracts in the inhibition of cell proliferation, induction of cell death, metabolic alterations and structural modifications in human breast (MCF-7, BT-20) and bone (MG-63, Saos-2) cancer cell lines. As a kind of control, non-tumorigenic cell lines of the breast (MCF-12A) and bone (POB) were included in the screening.

## Methods

### Plant material collection and identification

Four plants were employed in the present study. *Vincetoxicum arnottianum* and *Caccinia mancranthera* were collected from Baluchistan (Pakistan) and *Berberis orthobotrys* and *Onosma hispida* were collected from Gilgit-Baltistan (Pakistan) in 2014 (Table 1). The plants were identified by Dr. Sher Wali Khan and reference specimens were deposited at the Department of Biological Sciences, Karakoram International University, Pakistan.

### Preparation of extracts

Each plant sample including the aerial part of *V. arnottianum* (VSM), root (BORM) and fruit (BOFM) parts of *B. orthobotrys*, root (OHRM) and aerial (OHAM) parts of *O. hispida*, and the aerial part of *C. macranthera* (CMM) were air dried in shade and mechanically ground to fine powder. The finely-powdered material of each plant was soaked in methanol for several days and extracted. The dried methanolic extracts were obtained by removing the methanol by evaporation under reduced pressure. Furthermore, the fruit extract (BOFM) of *B. orthobotrys* was fractionated using solvent-solvent extraction and yielding n-hexane soluble oily substance (BO-23) and ethylacetate soluble oily substance (BO-5). Finally, eight samples i.e. VSM, BORM, BOFM, BO-5, BO-23, OHRM, OHAM and CMM were obtained and used for further study. Then, 50 mg of each dry sample was dissolved in 1 ml DMSO, EtOH or MeOH for the antitumor activity tests.

### Chemicals

For soaking and extraction purposes, the commercial grade solvents were used. For preparation of the samples for the antitumor activity, absolute ethanol, DMSO, and absolute methanol from Sigma Aldrich were employed.

### Cell lines, culturing and treatment conditions

Human osteosarcoma cell lines MG-63 (CRL-1427), Saos-2 (HTB-85) and human breast adenocarcinoma cell lines MCF-7 (ATCC: HTB-22), BT-20 (HTB-19) as well as non-tumorigenic human epithelial breast cell line MCF-12A (CRL-10782) were purchased from ATCC (http://www.lgcstandards-atcc.org/) under the given numbers. The human non-tumorigenic, primary osteoblast cells (POB) were chosen as control cells. Briefly, cells were isolated from the spongiosa of the femoral heads of patients undergoing primary total hip replacement. The samples were collected with patient agreement and approval by the Local Ethical Committee (registration number: A 2010-10). Human primary osteoblasts were already used and isolation procedure was already described [46]. Except for MCF-12A, all other cell lines and the primary POB cells were cultivated in Dulbecco’s modified Eagle’s medium (Invitrogen, Germany) with 10 % fetal bovine serum (PAN Biotech GmbH, Germany) and 1 % gentamycin (Ratiopharm, Germany). MCF-12A was grown in Dulbecco’s modified Eagle’s medium Ham’s F12 without phenol red (Invitrogen, Germany) containing 10 % horse serum (PAA Laboratories GmbH, Germany), the Mammary Epithelial Cell Growth Medium SupplementPack (PromoCell, Germany) including Bovine Pituitary Extract 0.004 nl/ml, Epidermal Growth Factor (recombinant human) 10 ng/ml, Insulin (recombinant human) 5 g/ml, Hydrocortisone 0.5 g/ml and 1 % gentamycin (Ratiopharm, Germany).

Prior treatment with the plant extract cells were adapted to phenol-red-free Dulbecco’s modified Eagle’s medium (PAA Laboratories GmbH, Germany) with 10 % charcoal stripped fetal bovine serum (PAN Biotech GmbH, Germany) for 48 h to avoid unspecific stimulation of endogenous hormones in the serum (assay medium). Treatment with plant extracts (final concentration 1, 10, 25, 50, and 100 μg/ml) was carried out for 48 h in assay medium. As negative control substance the vehicle DMSO, ethanol or methanol (0.1 %) was used in the same manner.

### Viability assay and calculation of IC_50_ values

MTS (3-(4, 5-dimethylthiazol-2-yl)-5-(3-carboxymethoxyphenyl)-2-(4-sulfophenyl)-2H-tetrazolium) assay to determine cell viability was performed according to manufactures protocol (CellTiter 96® AQueous One Solution Cell Proliferation Assay; Promega Corp., Madison, WI, USA). Briefly, cells were seeded in 96-well plates at a density of 2000 cells/well in 100 μl medium and left to attach for 24 h. Treatment with plant extracts at final concentrations of 1, 10, 25, 50 and 100 μg/ml was carried out as described previously [47]. In parallel, control approaches were carried out with medium only and 0.1 % of the solvent DMSO, EtOH or MeOH to calculate background absorbance. No background absorbance was obtained for the extracts and MTS in the absence of cells, as some extracts are capable of reducing the MTS. After an initial incubation for 24 h cells were assayed with MTS. Colorimetric changes were measured at 490 nm and raw data was transferred to Microsoft Excel and analyzed. At least 8 replicates corrected with the background absorbance were performed. Reduction of cell viability at each concentration was plotted as a dose response curve. The IC_50_ values were calculated using nonlinear regression to fit data to the dose–response.

### Cell cycle analysis

Proliferation alterations as well as apoptosis induction under the exposure of the plant extracts were estimated by cell cycle analysis via flow cytometry (FACS Calibur, BD Biosciences) after propidium iodide (Roche Diagnostics, IN, USA) staining (50 mg/ml) of the cells as already described [47, 48]. For data acquisition and histogram preparation, the software FlowJo version 7.6.5 (Tree Star; www.flowjo.com) was used. A minimum of 15,000 ungated events were recorded. Doublets and clumps were excluded by gating on the DNA pulse width versus pulse area displays. For statistical evaluation, the sum of cells in S- and G2/M-phase was defined as proliferative events and the sub-G1-peak of the histogram as apoptotic ones.

### Annexin V/PI apoptosis detection

Annexin-V detects the translocation of phosphatidylserine from the inner leaflets to the outer leaflets of the plasma membrane, which is a key feature of apoptotic cells, whereas PI detects necrotic cells with permeabilized plasma membrane. Labeling of early apoptotic and dead cells was performed according to the manufacturers’ instructions from the Alexa Fluor488 Annexin V/Dead Cell Apoptosis Kit (Thermo Fisher Scientific Inc., Germany). Cells were treated with 100 μg/ml plant extract for 48 h. After treatment detached as well as adherent cells were washed twice with cold PBS. The cell pellet was resuspended in 100 μl of annexin binding buffer at a density of 1 × 10^6^ cells per ml and incubated with 5 μl of Alexa488-conjugated Annexin-V and 5 μl of PI for 15 min at room temperature in the dark. 400 μl of 1× binding buffer was added to each sample tube, and the samples were immediately analyzed by flow cytometry. Histograms and statistics were designed with the software FlowJo Version 7.6.5.

### Microscopy

For bright field as well as fluorescence microscopic imaging, cells were seeded on glass cover slips and cultured for 24 h. After treatment with plant extracts bright field images were obtained using Axio Scope A1 microscope and the software AxioVision Imaging Software Release 4.8.2. (Carl Zeiss, Germany). For fluorescence imaging cell were fixed with 4 % paraformaldehyde for 15 min, followed by three washings with PBS and then permeabilized with 0.1 % Triton X-100 for 15 min. After carefully washing, cells were incubated with 100 μl 6.6 μM Alexa Fluor594 phalloidin (Invitrogen, Germany) for 60 min in the dark at room temperature, washed again, counterstained with DAPI (Roche Diagnostics GmbH, Germany) for 15 min. Finally, cell were washed four times with PBS and embedded in mounting medium. Lysosomes were labeled with LysoTracker® Green DND-26 (Molecular Probes, Carlsbad, CA, USA) following the protocol supplied. The other cell compartments: mitochondria (MitoTracker® Mitochondrion-Selective Probes Green FM), Golgi complex (BODIPY® FL C5-ceramide complexed to BSA), endoplasmic reticulum (ER-Tracker™ Green BODIPY® FL glibenclamide), neutral lipids (4,4-difluoro-1,3,5,7,8-pentamethyl-4-bora-3a,4a-diaza-s-indacene BODIPY® 493/503), all from Molecular Probes, Germany were labeled following the manufactures’ instructions. All fluorescence signals were investigated with an inverted confocal laser scanning microscope (LSM780, Carl Zeiss, Germany) equipped with a helium/neon-ion laser and a ZEISS 63 × oil immersion objectives. The confocal images (1024 × 1024 pixel) were optimized using the ZEN software (Carl Zeiss, Germany).

### Scanning electron microscopy

For scanning electron microscopy (SEM) cells grown on glass cover slips were fixed with 2 % glutaraldehyde and 1 % PFA in 0.1 M phosphate buffer pH 7.3. After washes in 0.1 M phosphate buffer the cells were dehydrated with a graded series of ethanol and were processed for critical point drying using CO_2_ as intermedium (Emitech K850 critical point dryer, Emitech Ltd. Ashford, UK). The cover slips were mounted on SEM stubs with adhesive carbon tape (Plano, Wetzlar, Germany) and sputter-coated with a gold layer (approximately 15–20 nm thickness) using a Bal-Tec SCD004 sputter coater (Balzers Union Ltd., Balzers, Liechtenstein). Specimens were viewed in a field-emission SEM operated at 5 kV (Merlin VP compact, Carl Zeiss Microscopy, Jena, Germany) and images with a size of 1024 x 768 pixels were recorded. Morphometric measurements of cell body axis length and width were taken with the free line measurement tool on calibrated pictures imported into iTEM imaging software (Olympus Soft Imaging Solutions, Münster, Germany).

### Mitochondrial O_2_-consumption

Mitochondrial O_2_-consumption as a measure for respiratory activity was determined by the Bionas® 2500 analyzing system combined with the metabolic chip Bionas DisocveryTM SC1000 equipped with Clark-type oxygen sensors. Prior experiments, chips were cleaned with 70 % ethanol for 10 min, washed with PBS and were adapted to the measurement medium for 5 min. Measurement medium was composed of DMEM without NaHCO3 (Invitrogen, Germany), 0.1 % charcoal stripped fetal bovine serum (PAN Biotech GmbH, Germany) and 1 % gentamycin (Ratiopharm, Germany), pH value 7.4 and sterile filtered. On each chip 2x10^6^ cells were seeded and let them adhere over night at 37 °C and in 5 % CO_2_ so that 80 % sub-confluence on the sensor chips was reached. Bionas measurements were carried out with a pump rate of 56 ml/min [49]. After an adaption phase of 2 h to the new culture conditions, extracellular oxygen consumption of MG-63 cells after application of 25 μg/ml BORM or VSM was measured continuously for 20 h. Thereafter the recovery status (measurement medium without plant extracts) of the cells was monitored for additional 24 h. Data sets were evaluated and normalized with the software Bionas15002 Data analyzerV1.07.

### Migration and invasion

Influence on migration was conducted on MG-63 cells, pre-incubated in assay medium for 48 h adaption in 6-well plates (Greiner, Germany). A scratch wound was made by Ibidi culture inserts (μ-Dish 35 mm; Ibidi GmbH, Martinsried, Germany) following the instructors recommendations. When cell layers reached confluence, the culture insert was removed and cells were treated with VSM (25–50 μg/ml) extract or control (vehicle, DMSO). Gap closure was analyzed as described previously [50]. Cell invasion assay was performed with the CytoSelect™ 24 –Well Cell Invasion Assay (Basement Membrane, Fluorometric Format) from Cell Biolabs, Inc., CA, USA. Briefly, 1x10^6^ MG-63 cells with the plant extracts were seeded in the membrane insert for 48 h. Fluorescence of invaded cells was counted with a plate reader at 480/520 nm.

### Western blotting procedure

The general steps of the Western blot procedure have been described previously [49]. Briefly, after treatment with the plant extracts VSM and BORM for at least 48 h the cells were trypsinized, washed with PBS and lysed in ice-cold lysis buffer (Bio-Plex Cell Lysis Kit, Bio-Rad, USA). After SDS-PAGE, protein content per lane as well separation quality was controlled with the Criterion Stain FreeTM gel imaging system (Bio-Rad, Germany). For protein detection primary antibodies (PCNA: sc-56, from Santa Cruz, USA; BCL-2: B3170, from Sigma) were incubated overnight at 4 °C followed by labeling with a horseradish peroxidase (HPR)-conjugated secondary antibody (Dako, Glostrup, Denmark) for 1 h at room temperature. Protein signals were visualized by using SuperSignal West Femto Chemiluminescent Substrate (Pierce Biotechnology, Rockford, USA). Band intensity was analyzed densitometrically with the Molecular Imager ChemiDoc XRS and Image Lab 3.0.1 software (Bio-Rad, USA). Protein detection was repeated at least three times with individually prepared cell lysates from independently passaged cells.

### Statistical analysis

Every experiment was replicated three times with individually passaged cells and data sets were expressed as means ± standard deviations (SD). Statistical significance was determined by the unpaired one-way ANOVA or *t*-test (***P < 0.001, **P < 0.005, *P < 0.05).

## Results

### Initial screening on cell viability

**Fig. 1:**
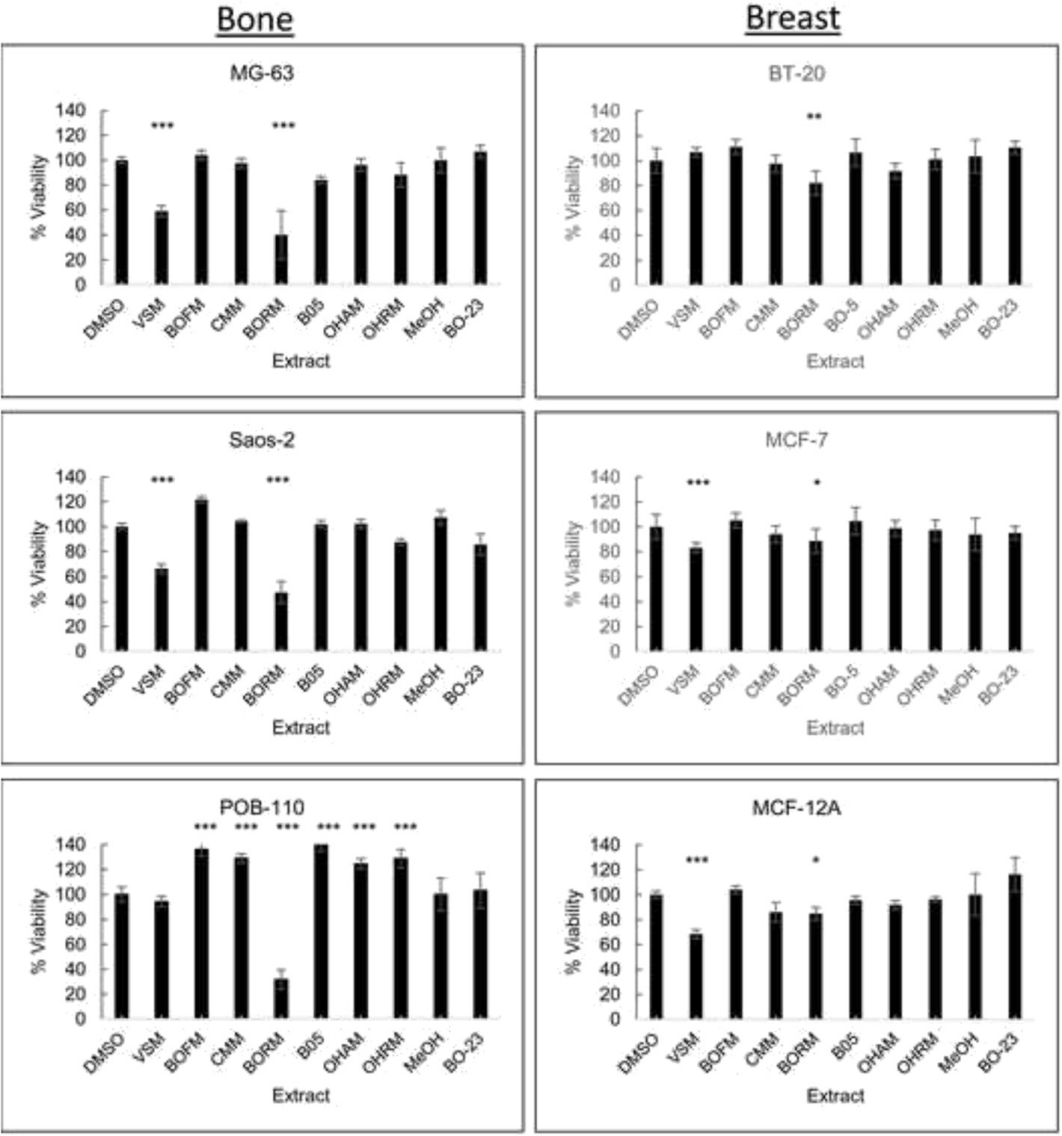
Determination of cell viability. Measurement of cell viability via MTS assay after exposure to 50 μg/ml plant extract for 48 h on respective bone (MG-63, Saos-2) and breast (BT-20, MCF-7) cancer cell lines in comparison with primary osteoblasts (POB) and non-tumorigenic mammary epithelial cells (MCF-12A). As control treatment the vehicle DMSO and MeOH were used at a final concentration of 0.1 % (w/v). Samples were compared using one-way ANOVA. Error bars indicated mean ± SD, *n* = 8, ***P < 0.001, **P < 0.01, *P < 0.5, significantly different compared to control

To evaluate the anticancer properties of the Pakistani plant extracts two bone (MG-63, Saos-2) and two breast (BT-20, MCF-7) cancer cell lines in comparison with primary osteoblasts (POB-110) and non-tumorigenic mammary epithelial cells (MCF-12A) were selected. The osteosarcoma cell line MG-63 represents an early osteoblastic type while Saos-2 cells exhibited the most mature osteoblastic phenotype [51]. The breast cancer cell line MCF-7 represents the luminal, estrogen and progesterone receptor-positive subtype whereas BT-20 cells are invasive, triple-negative breast cancer cells [52]. For the initial screening all cells were treated with 50 μg/ml of each plant extract for 48 h and cell viability was measured right after (Fig. 1). The extracts VSM and BORM caused the greatest significant reduction (40–60 %) of cell viability in the osteosarcoma cell lines MG-63 and Saos-2. On primary osteoblast cells (POB-110) VSM induced only a slight decrease in cell viability while BORM lowered the viability up to 80 %. Beside VSM and BORM, only the treatment with BO-5 on MG-63 cells as well as OHRM on Saos-2 cell revealed a significant viability decrease of 10–15 %. These results illustrate that the VSM extract has anticancer potential on bone cancer cells, since it selectively reduced the vitality of osteosarcoma cells and only exerts a minimal effect on the primary osteoblasts. Similar results were achieved for the treatment of the breast cancer cell lines. On BT-20, hormone-independent and invasive carcinoma cells, only the BORM extract caused a slight viability reduction of approximately 20 %. On MCF-7 cells, VSM as well as BORM induced decreased vitality rate in a range of 10–20 %. This vitality reduction was also measured on the non-tumorigenic control cell line MCF-12A indicating that the extracts VSM and BORM displayed strong cytotoxic effects which will be analyzed in the next sections.

### Influence on cell cycle phases of MG-63 osteosarcoma cells

**Fig. 2:**
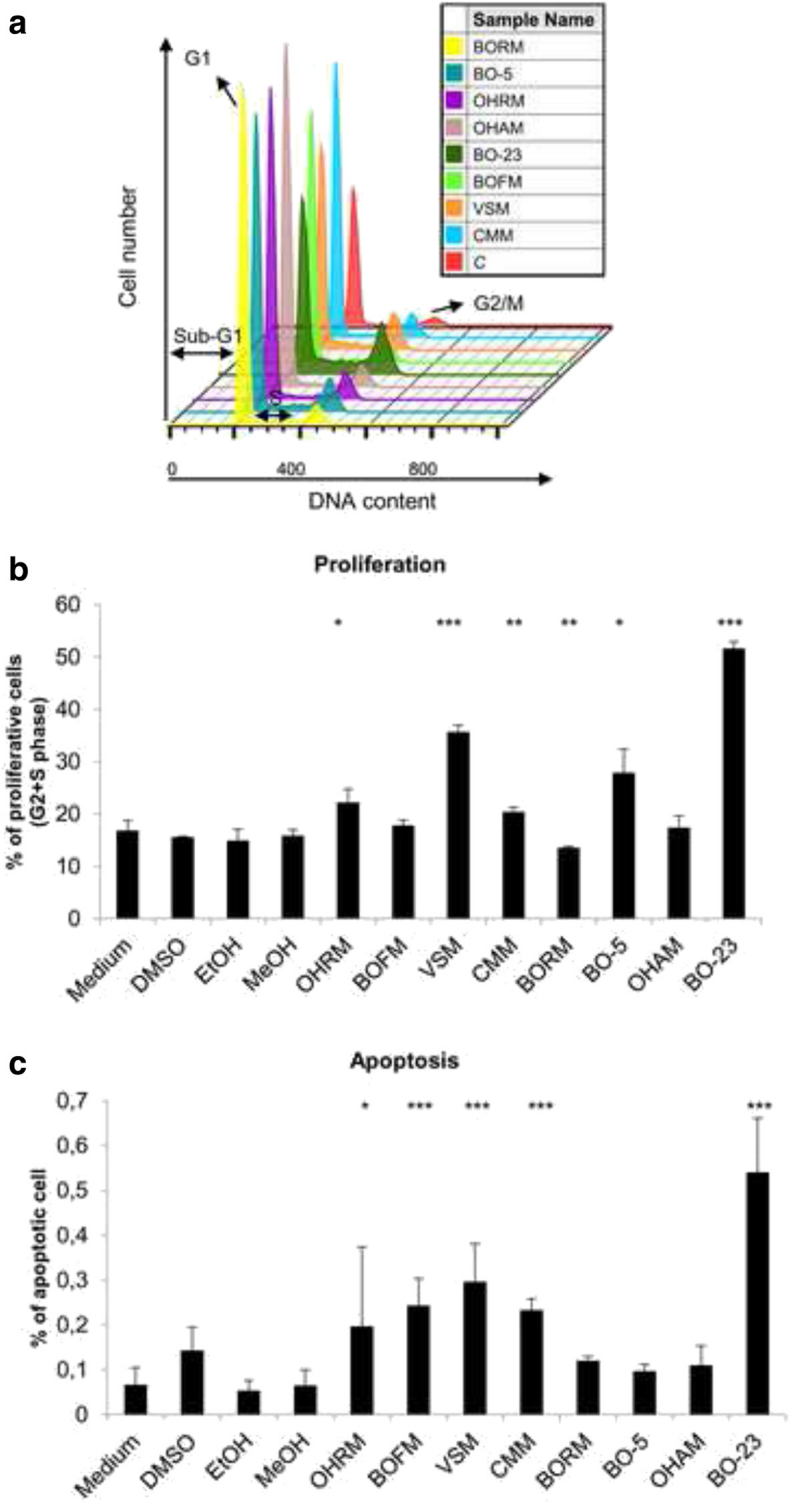
Cell cycle alterations of MG-63 cells. **a** Histogram of the cell cycle distribution of MG-63 cells after treatment with the control substance (**c**) and the eight samples of plant extracts at a concentration of 50 μg/ml for 48 h. G1, S, G2/M and sub-G1 phases are marked with black arrows. Represented were the most prominent samples of 3–5 individual replicates. **b** Calculation of proliferation after treatment with the vehicle (DMSO, EtOH, MeOH; equates to 100 %) and the plant extracts at a concentration of 50 μg/ml for 48 h. As proliferative phases the sum of S and G2/M phases were calculated in percentages. **c** As apoptotic fraction the sub G1-peak was measured. (mean ± SD, *n* = 3–5, ***P < 0.001, **P < 0.01, *P < 0.5, significantly different compared to control, one-way ANOVA)

Besides the vitality measurements, the influence on cell growth and the induction of apoptosis are important parameters to evaluate the respective anticancer properties of the plant extracts. Therefore, cell cycle analyses were performed to determine the influence on the proliferation behavior (G2/M + S phase) and apoptosis initiation by DNA strand breaks (sub G1 phase), simultaneously (Fig. 2). Exemplarily, for all cell lines used, Fig. 2 demonstrates the DNA histogram (Fig. 2a), proliferation alterations (Fig. 2b) and the number of apoptotic cells (Fig. 2c) after 48 h treatment with 50 μg/ml plant extract on MG-63 osteosarcoma cell line.

Both, in the histogram and in the proliferation diagram is clearly evident that the extracts OHRM, VSM, BO-5 and BO-23 accelerate the cell number in the proliferative phases G2 and M. Only BORM caused a slight significant reduction in the proliferative phases (20 %). The number of apoptotic cells increased after treatment with 50 μg/ml of BOFM, VSM, CMM and BO-23, significantly. In summary, some of the plant extracts display an effect on the proliferative phases G2/M and S, but do not affect the sub-G1 phase. The DNA-histograms of the control treatments with medium, DMSO, EtOH and MeOH (final concentration: 0.1 μg/ml) are given in Additional file 1: Figure S1, showing no alterations in the cell cycle phases.

### Concentration dependent effects of BORM and VSM

**Fig. 3:**
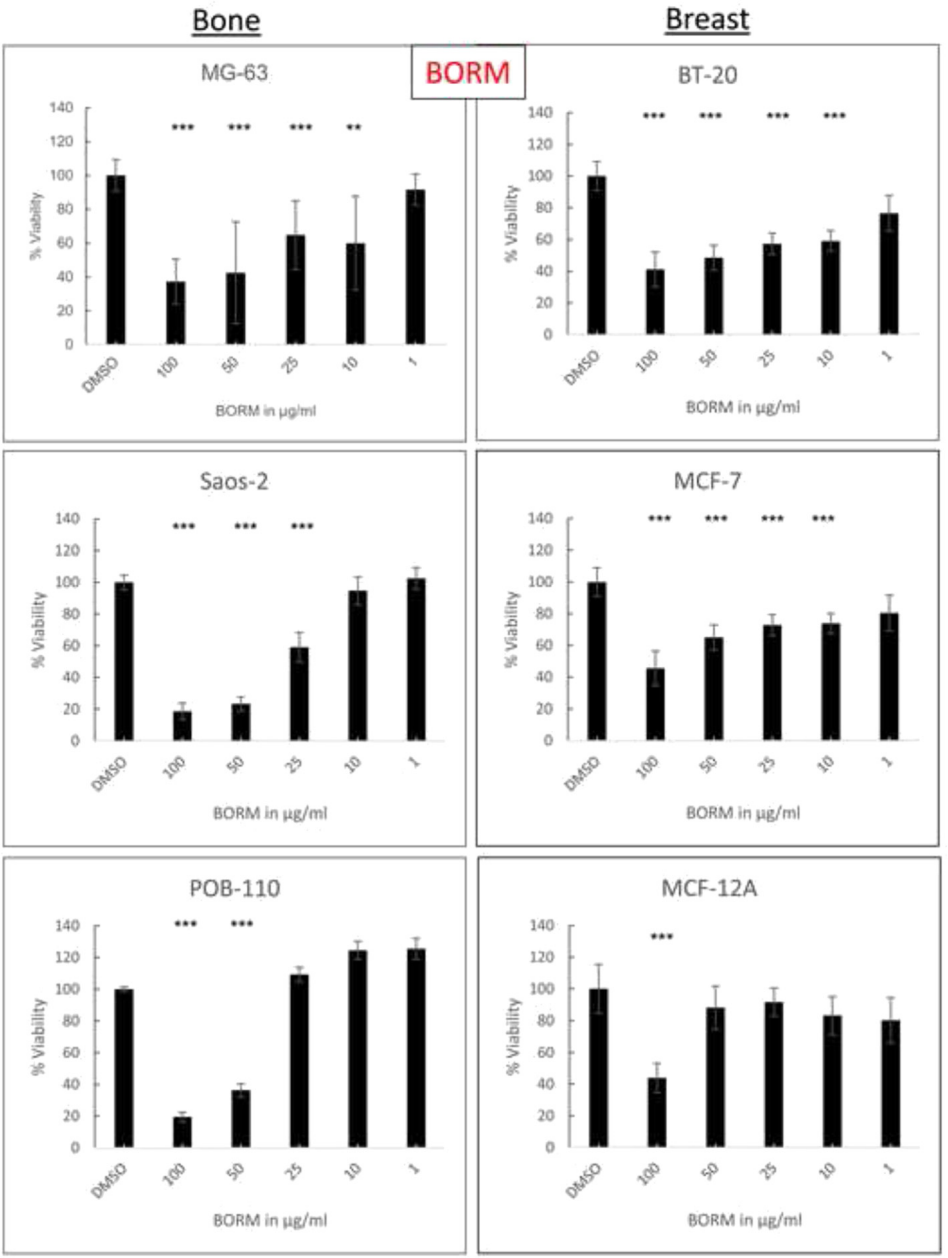
Concentration dependent effects of BORM. Cell viability measurement in concentration series of BORM in a range from 1 to 100 μg/ml after exposure of 50 μg/ml plant extract for 48 h on respective bone (MG-63, Saos-2) and breast (BT-20, MCF-7) cancer cell lines in comparison with primary osteoblasts (POB) and non-tumorigenic mammary epithelial cells (MCF-12A). The solvent DMSO was used as a negative control. Mean ± SD, *n* = 6–8, ***P < 0.001, **P < 0.01, *P < 0.5, significantly different compared to control, one-way ANOVA

**Fig. 4:**
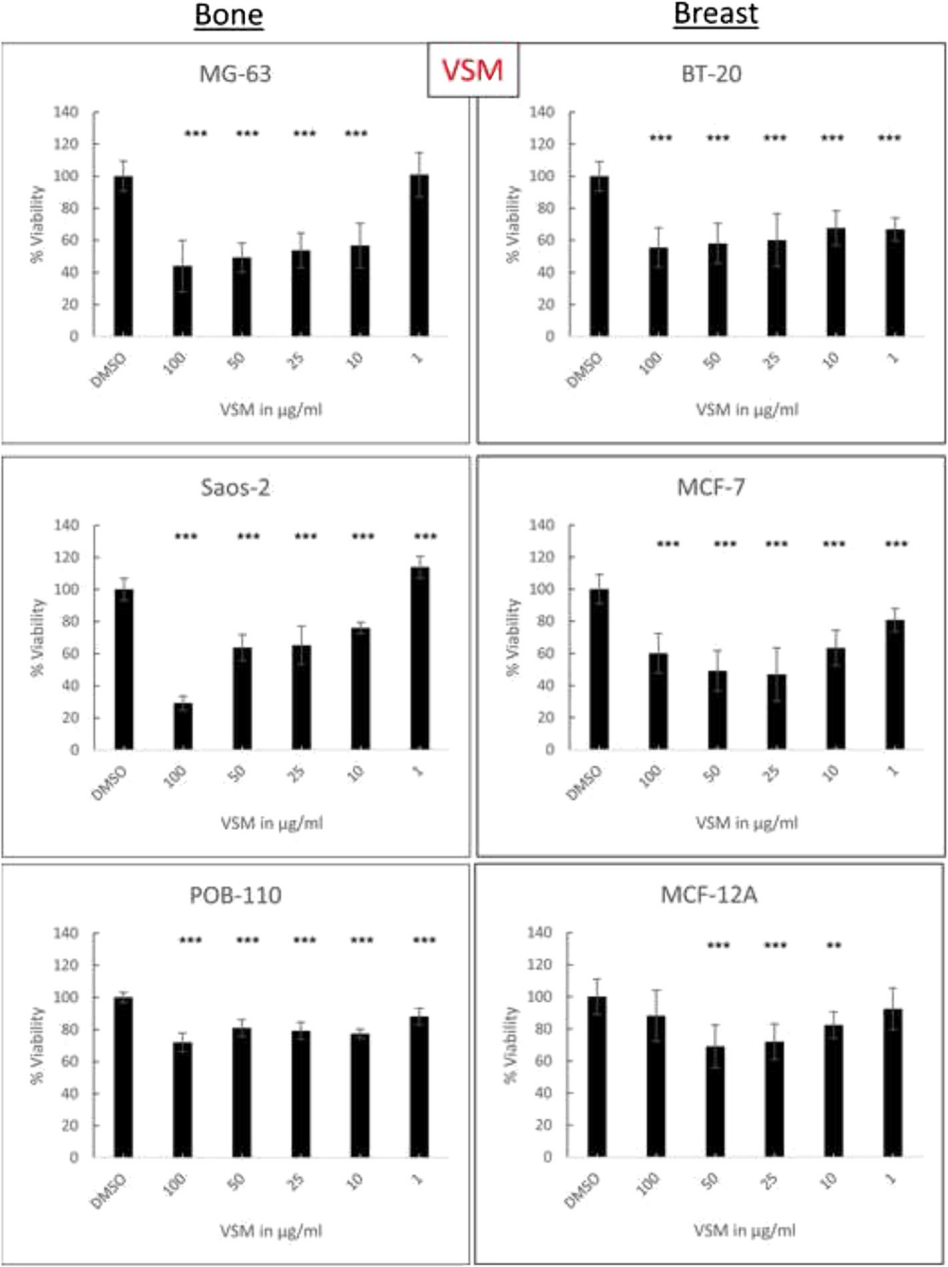
Concentration dependent effects of VSM. Cell viability measurement in concentration series of VSM in a range from 1 to 100 μg/ml after exposure of 50 μg/ml plant extract for 48 h on respective bone (MG-63, Saos-2) and breast (BT-20, MCF-7) cancer cell lines in comparison with primary osteoblasts (POB) and non-tumorigenic mammary epithelial cells (MCF-12A). The solvent DMSO was used as a negative control. Mean ± SD, *n* = 6–8, ***P < 0.001, **P < 0.01, *P < 0.5, significantly different compared to control, one-way ANOVA

As BORM and VSM caused the most significant effects on all bone and breast cancer cell lines, both extracts were examined in concentration series ranging from 0.1 to 100 μg/ml to evaluate the concentration dependent effects (Figs. 3 and 4) on cell viability and to calculate the IC_50_ values (Table 2). Therefore, all the cell lines were used in non-confluent cell cultures (confluence at treatment beginning: 60–80 %).

**Table 2:**
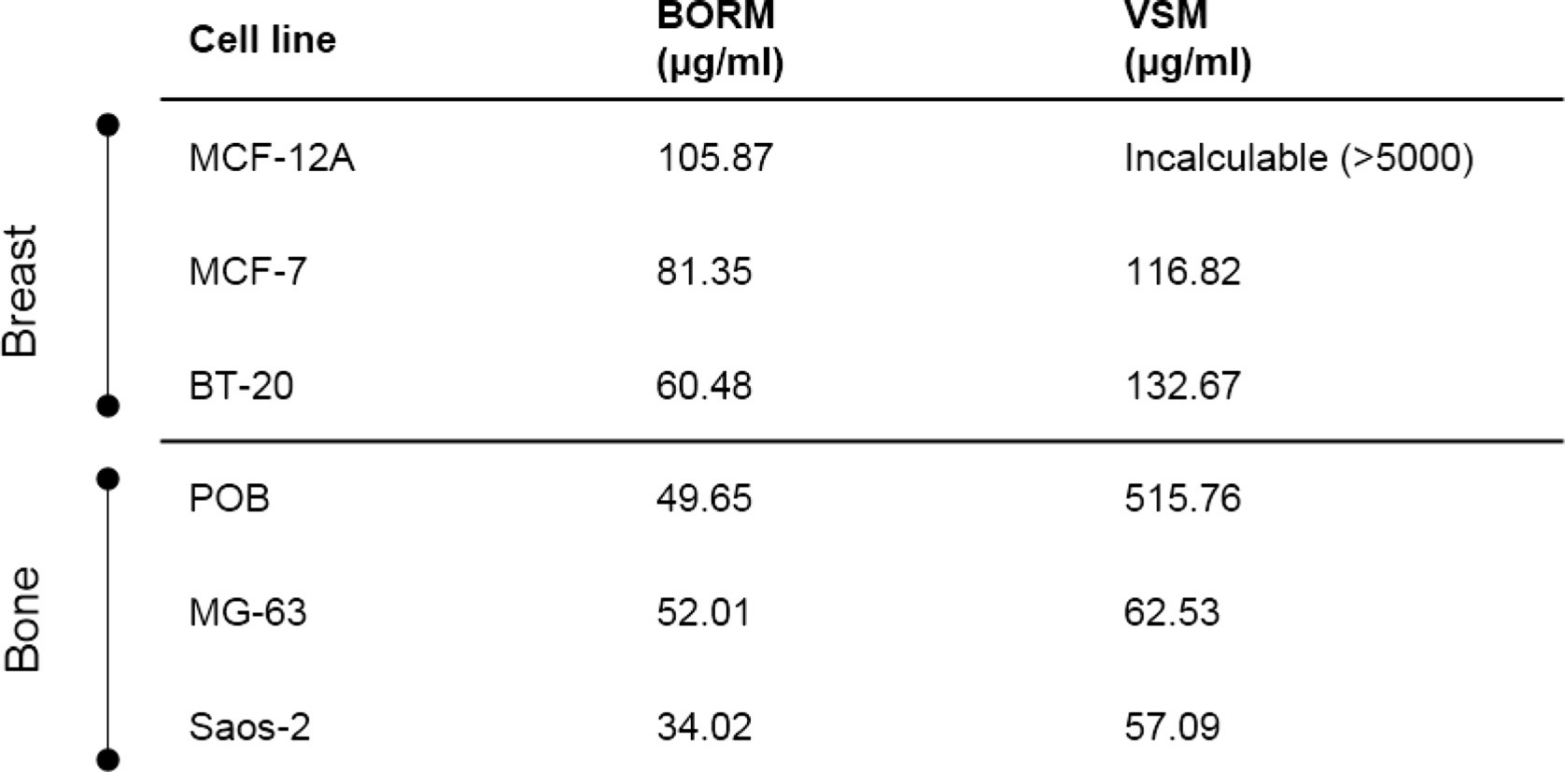
Overview of the calculated IC_50_ values. IC_50_ values of the plant extracts BORM and VSM on the bone and breast cancer cell lines in comparison with the non-tumorigenic control cells determined by viability measurements after 48 h treatment

BORM induced linear concentration dependent effects on the osteosarcoma cell lines MG-63 and Saos-2 as well as on the breast cancer cell lines BT-20 and MCF-7 (Fig. 3). The highest concentration of 100 μg/ml reduced the cell viability by 60–80 % in all tumorigenic and control cells. However, in comparison with cancer cells, the control cells POB-110 and MCF-12A were not that strongly affected by BORM in concentrations below 50 μg/ml. This is confirmed by the calculation of the IC_50_ values: BORM exhibited the lowest values for BT-20 and Saos-2 (~60 and 34 μg/ml), indicating a pronounced cytotoxicity (Table 2). In contrast, VSM caused only a linear concentration dependent effect on the bone cancer cell lines MG-63 and Saos-2 leading to IC_50_ values of 62.53 and 57.09 μg/ml, respectively. The IC_50_ value for the control primary osteoblasts POB was significantly higher (515.76 μg/ml) indicating that VSM mediates a stronger cytotoxic impact on the bone cancer cell lines. The vitality of the non-tumorigenic mammary epithelial cell line MCF-12A was only minimally affected by VSM leading to a no calculable IC_50_ value. On the other hand VSM displayed moderate IC_50_ values for BT-20 and MCF-7 (132.67, 116.82). These results illustrate that the plant extract VSM has anti-tumor potential, primarily because the viability of the cancer cells is reduced and the influence on the non-tumorigenic control cells is low.

**Fig. 5:**
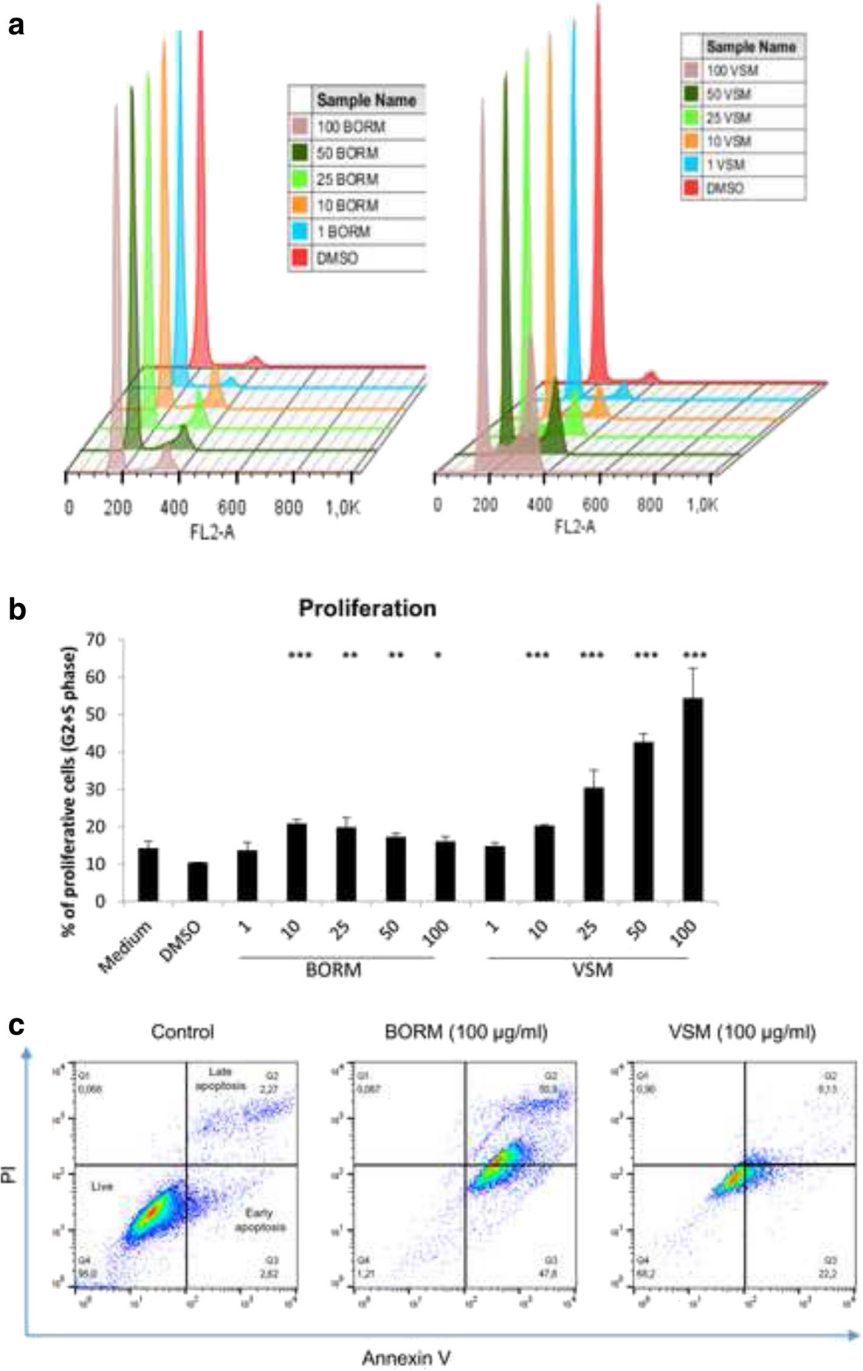
Cell cycle phases, proliferation and apoptosis events. Cell cycle analysis of VSM and BORM treated MG-63 cells (48 h) in concentration series ranging from 1 to 100 μg/ml in comparison with the control treatment (DMSO). **a** Histograms of the cell cycle distribution. **b** Calculation of proliferation. As proliferative phases the sum of S and G2/M phases were calculated in percentages. Mean ± SD, *n* = 3–4, ***P < 0.001, **P < 0.01, *P < 0.5, significantly different compared to control, one-way ANOVA. **c** Annexin V/PI staining to label living, early and late apoptotic events after treatment with the control (DMSO), 100 μg/ml BORM or VSM measured via flow cytometry

These dose dependent effects were verified by cell cycle measurements (Fig. 5a, b) and apoptosis detection (Fig. 5c) on the osteosarcoma cell line MG-63, exemplarily. In comparison with the control treatments BORM induced a slight increase of the proliferative phase G2/M, starting at a concentration of 10 μg/ml. In contrast, the VSM extract caused a linear, concentration-dependent increase in the proliferative phase G2/M and S, indicating for a G2-arrest. At a concentration of 100 μg/ml VSM more than half of the analyzed cells were detected in the G2/M phase. To verify the apoptosis induction an Annexin V/PI staining was performed (Fig. 5c, Additional file 2: Figure S2). 100 μg/ml BORM induced an increase in early and late apoptotic events up to 50 %. By contrast, VSM caused a slight shift in early apoptotic events (~ 20 %). All together, these results suggest that BORM and VSM exhibit anti-tumorigenic potential. The precise mode of action is to be analyzed in the next chapters.

### Morphological, cytoskeletal and cell compartment alterations

**Fig. 6:**
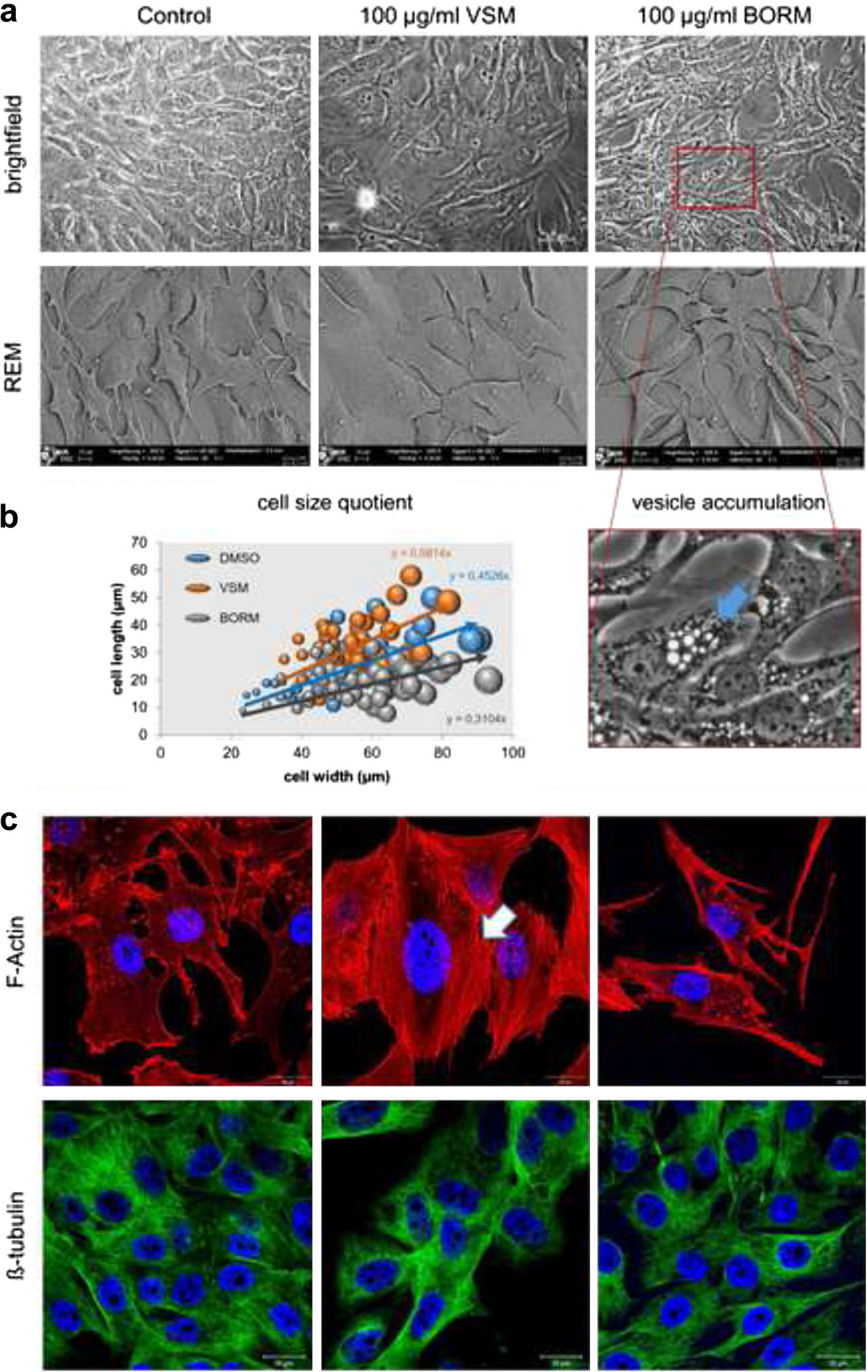
Morphological and cytoskeletal alterations of MG-63 cells analyzed by different microscopic techniques. **a** Morphological alterations of MG-63 cells after treatment with DMSO (control) or 100 μg/ml VSM and BORM by bright field imaging (*upper panel*) and scanning electron microscopy (*lower panel*). **b** Calculation of the cell size quotients by length and width measurements of the cells in the scanning electron micrographs (*left*). *n* = 30. Enlarged section of BORM treated MG-63 cells. Distinctly, the vesicles accumulation can be detected around cell nucleus (*right*). **c** Laser scanning microscopic images of F-actin (*red*) and ß-tubulin (*green*) stained MG-63 cells. Cells were counterstained with Hoechst to label the cell nuclei (*blue*). Notably, VSM induced an increased Actin stress fiber formation through the entire cell, leading to a greater cell surface area. In contrast, treatment with BORM caused an increased production of vesicle-like structures and a spindle-shaped cell shape. Magnification bars = 20 μm

**Fig. 7:**
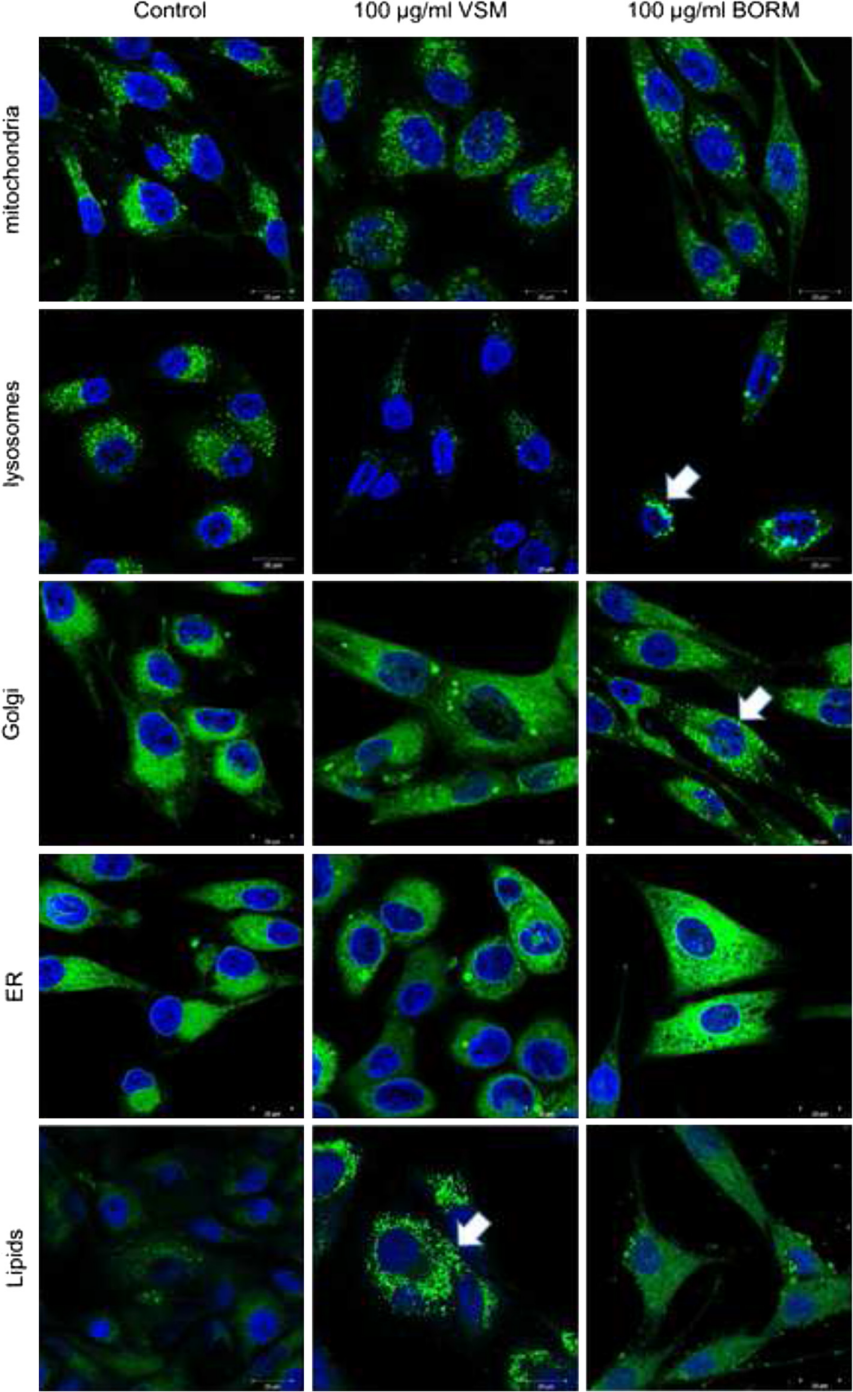
Structural cell compartment alterations of MG-63 cells. Laser scanning microscopic analysis of cell compartments (mitochondria, lysosomes, Golgi apparatus, endoplasmic reticulum, and neutral lipids) within MG-63 cells treated with 100 μg/ml BORM or VSM in comparison to the control (DMSO). Cells were counterstained with Hoechst to label the cell nuclei (*blue*). Notably, exposure to VSM caused a reduced production of lysosomes and a strong increase of neutral lipids. In contrast, BORM treatment revealed an accumulation and augmentation of lysosomes, stronger granularization and formation of Golgi vesicles and a diffuse distribution of neutral lipids

To characterize morphological and cytoskeletal changes (F-actin and ß-tubulin), MG-63 cells were cultured in the presence of BORM and VSM (0.1–100 μg/ml) or vehicle control (0.1 % DMSO) for 48 h, and monitored by bright field, scanning electron and laser scanning microscopy (Fig. 6; Additional file 3: Figure S3, Additional file 4: Figure S4). Under control conditions MG-63 cells form a confluent monolayer with the typical fibroblast-like cell structure (Fig. 6a). Confocal imaging revealed that untreated MG-63 cells possess cortical actin, some well-defined stress fibers, and cell polarity as shown by the presence of lamellipodia (Fig. 6c). VSM caused a remarkable change in cell shape: cells are wider and have a larger cell surface (Fig. 6b). Furthermore, a reduced formation of the cortical cytoskeleton and a solid reinforcement of actin stress fibers through the entire cell area are visible. The stress fibers are much longer, thicker and stabilize the entire cell, so that the cell contacts are partially broken. Exposure to BORM resulted in a strong increase of vesicles in the cell nucleus environment, observed both in the bright field image as well as in the F-actin staining (Fig. 6a-c). The cells are much more stretched and spindle-shaped, resulting in a smaller cell area (Fig. 6b). The formation of the actin fibers as well as the distribution of cortical actin did not change, substantially. The formation of tubulins is neither changed after treatment with BORM nor VSM. The increased formation of vesicles after exposure to BORM as well as the strengthening of the actin skeleton after treatment with VSM can be due to metabolic alteration or cell compartment disorders. Therefore, primary cell compartment alterations were monitored by live cell imaging (Fig. 7). The number and distribution of mitochondria as well as the appearance of the endoplasmic reticulum remained almost unchanged. But in contrast, the exposure to BORM revealed an accumulation and augmentation of lysosomes while treatment with VSM reduced the amount of lysosomes, significantly. Already at a starting concentration of 1 μg/ml the number of lysosomes increased, and reached the highest number at a concentration of 25 μg/ml BORM. Higher concentrations of BORN did not further elevate the amount of lysosomes but caused a merger of lysosomes so that the size increased up to the 3–5 fold (Additional file 5: Figure S5). Similarly, treatment with BORM caused a change of the Golgi apparatus: a stronger granularisation and formation of Golgi vesicles can be observed. The staining of neutral lipids, which was only very slightly visible in the untreated cells, was strongly upregulated after VSM exposure. Many small dots of lipids could be verified around the nucleus and in the cytoplasmic area. In contrast, treatment with BORM resulted in a diffuse distribution of neutral lipids in the cell without any specific dot distribution. Up to this state, it can be concluded that BORM and VSM mediate cytotoxic effect by affecting different metabolic pathways. To discuss this more profound, various metabolic and motility-specific investigations were carried out.

### Influence on O_2_ consumption, motility and selected protein marker expression

**Fig. 8:**
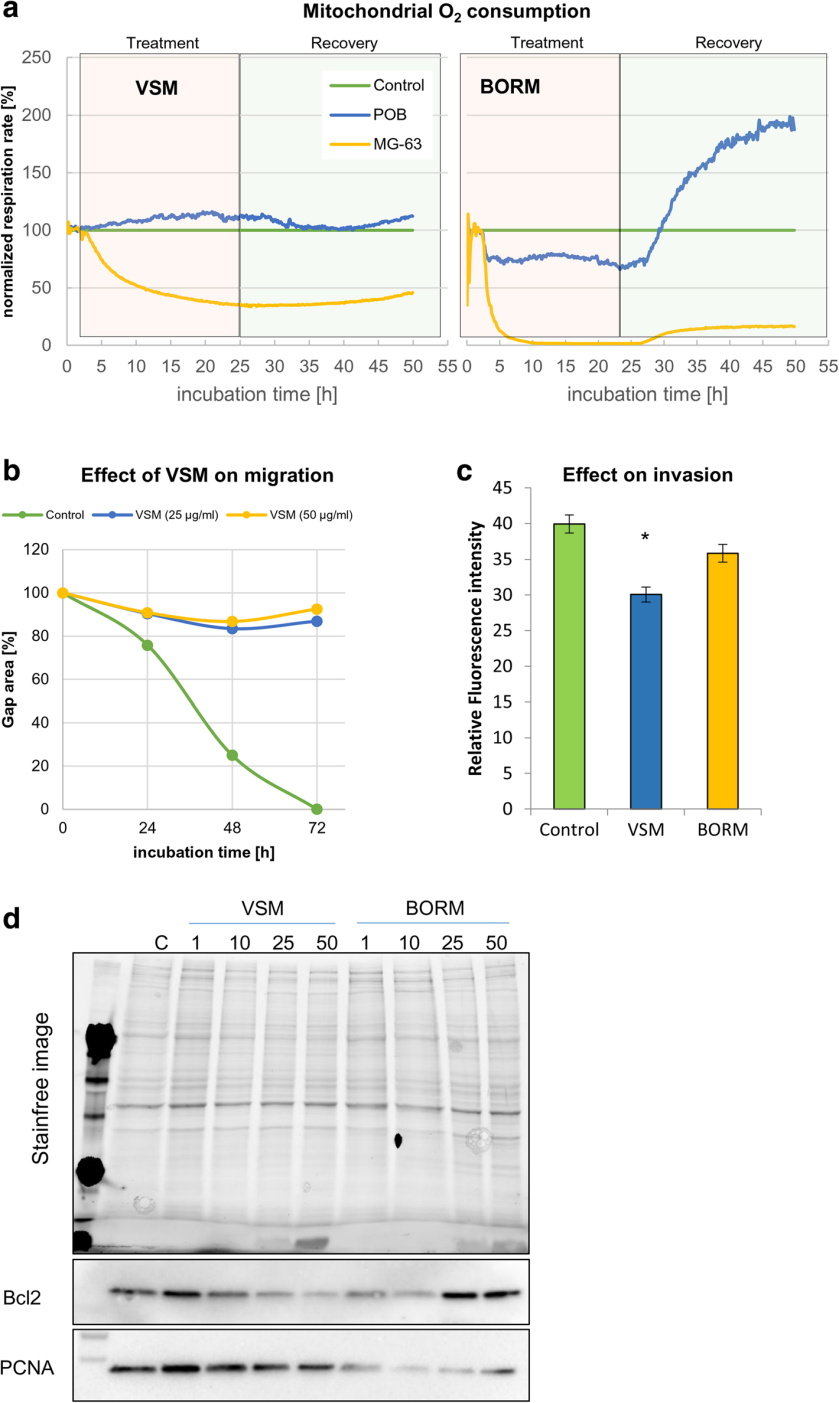
Influence on O_2_ consumption, motility and selected protein marker expression. **a** Mitochondrial O_2_ consumption (respiration) in MG-63 cells and primary osteoblasts (POB) during exposure to 25 μg/ml VSM or BORM in comparison to the control (which was set to 100 %) determined by the Bionas® 2500 analyzing system combined with the metabolic chip Bionas DisocveryTM SC1000 equipped with Clark-type oxygen sensors. Both BORM and VSM reduce the respiration rate dramatically (99 and 60 % reduction, respectively) in tumorigenic osteoblasts. **b** Effect of VSM (25, 50 μg/ml) on MG-63 migration behavior in comparison to the control treatment (DMSO) in a 72 h time period in a wound healing assay (raw data available in Additional file 7: Figure S7). Notably, exposure to VSM prevents migration of MG-63 cells so that the cell lawn cannot be closed. **c** Invasion assay of BORM and VSM treated MG-63 cells. Only VSM reduced the invasion capacity, significantly. Mean ± SD, *n* = 3, *P < 0.5, significantly different compared to control, unpaired *t*-test. **d** Western blot analysis of proliferation (PCNA) and apoptosis (Bcl-2) marker expression in VSM or BORM treated (concentration series 1–50 μg/ml) MG-63 cells in comparison to the control (**c**). The stain free image of the polyacrylamide gel functions as loading control

To examine the cell specific mode of action of the two plant extracts, the influence on cell metabolism was investigated, firstly. Therefore, live cell monitoring of three metabolic parameters (cell impedance, O_2_-consumption and extracellular acidification) was performed (Fig. 8a, Additional file 6: Figure S6). Both, the treatment with VSM and BORM for 24 h resulted in a strong reduction (VSM: 75 %, BORM: 99 %) of the mitochondrial O_2_-consumption in MG-63 cells (Fig. 8a). This decrease in respiratory capacity cannot be reverted after the discontinuation of the plant extracts. In contrast, the effect on primary osteoblasts (POB) was different: VSM did not alter the respiration capacity; BORM induced a slight decrease in O_2_-consumption (~ 20 %) which could not only be reverted but enhanced up to 100 % after discontinuation of the plant extract. Beside this strong effect on cellular energy metabolism, the effect on cell motility was investigated. Because VSM induced an increased formation of stress fibers (Fig. 6), the influence on cell migration and invasion was determined (Fig. 8b, c). Concentrations of 25–50 μg/ml VSM decreased the migratory activity (90 %) and the invasiveness (35 %) of MG-63 cells. BORM did not alter the cell motility, significantly but induced apoptotic signals by enhanced BCL-2 expression and proliferation reduction by PCNA repression (Fig. 8d).

## Discussion

In this study, eight samples from four Pakistani plant extracts were evaluated for their potential as anticancer agents in selected human bone and breast cancer cell lines in comparison with non-tumorigenic control cells via cell viability measurements, cell cycle analysis, live cell imaging and monitoring of metabolic as well as motility features. After the first initial screening, BORM and VSM revealed the highest potential with regard to its antitumor activity. Both extracts caused a significant reduction of cell viability in the breast and bone cancer cells. However, BORM also induced a strong reduction of cell viability in the primary osteoblasts (POB), as well as VSM lowered the cell vitality in the non-tumorigenic breast cell line MCF-12A. But, VSM caused no negative influence on POBs wherein the bone cancer cell lines were strongly influenced (Fig. 1). These results suggest that the therapeutic use of VSM particularly for the treatment of bone cancer would be possible. For the treatment of breast cancer the BORM extract may be suitable on the basis of the vitality studies. Because BORM caused only a marginal effect on the vitality of the control cell line MCF-12A and induces a significant vitality reduction in both, the estrogen receptor-positive breast cancer cell line MCF-7 and in the triple-negative cell line BT-20.

Subsequent cell cycle analysis revealed a substantial increase of the proliferative phases G2/M and S after exposure to 50 μg/mg VSM whereas BORM slightly lowered the proliferation (Fig. 2a-b; exemplarily illustrated at the bone cancer cell line MG-63). Although VSM especially increases the G2/M phase in MG-63 cells, a simultaneous increase in DNA strand breaks, to be mentioned in the sub-G1 phase (Fig. 2c), could be observed. This suggests that the VSM extract induces apoptotic changes which are often associated with elevated proliferation rates in order to obtain the cell layer. Another possibility is a G2/M arrest of the cell population similar to the effect of paclitaxel which stabilizes tubulin polymerization resulting in arrest in mitosis and apoptotic cell death [53].

So far, the obtained results imply that the extracts VSM and BORM mediate different cellular responses which lead to cytotoxic events. In order to identify these cellular mechanisms, dose-response curves were created first (Figs. 3 and 4). From these curves it can be concluded that both extracts exert concentration-dependent effects on both, breast as well as bone cancer cells. The calculated IC_50_ values (Table 2) show that VSM primarily affects the bone cancer cells and only minimally impaired the vitality of healthy osteoblasts. The IC_50_ values of BORM illustrate that this extract reduces the vitality of the breast cancer cell, predominantly. For the non-tumorigenic control cell line MCF-12A a considerably higher IC_50_ value was determined.

However, bright field, scanning electron and laser scanning microscopy observations revealed morphological and structural alterations of MG-63 osteoblastic cells after exposure to 100 μg/ml VSM or BORM (Fig. 6). In comparison to the control, VSM treated MG-63 cells exhibit a prolonged shape accompanied with reduced cell-cell contacts. F-actin staining revealed a strong induction of stress fiber formation through the entire cells. Along with the reduced cell viability, the mediated G2/M arrest in the cell cycle phases and increased actin fiber formation can be assumed that the VSM extract causes a stabilization of the tumor cells, thus causing the cytotoxic properties. In contrast, the BORM extract promotes the formation of vesicle-like structures in the cell which can be due to a stimulation of the lysosomal activity or aggregation of lysosomal vesicles. Even at low concentration (1–10 μg/ml BORM) an increased formation of lysosomes was observed (Fig. 7). The higher the BORM concentrations, the greater the expansion of the lysosomal compartments. At the highest concentration (100 μg/ml) the lysosomes are large clusters around the nucleus (Fig. 7). This means that the cytotoxic effect of BORM is due to the activation of lysosomes which can selectively activate programmed cell death [54]. Briefly, lysosomal ROS generation can cause lysosomal membrane permeabilization, whereby lysosomal cathepsins, as well as other hydrolytic enzymes, are released from the lysosomal lumen to the cytosol, and can trigger programmed cell death [55, 56]. In addition, BORM caused a stronger granularisation and formation of Golgi vesicles as well as a diffuse distribution of neutral lipids. This is not surprising, because it is thought that the reservoir of chemicals in the lysosome can be ‘topped up’ by supplies from the Golgi apparatus. The chemicals are manufactured in the endoplasmic reticulum, modified in the Golgi apparatus and transported to the lysosomes in vesicles (sealed droplets). Modification in the Golgi apparatus includes ‘destination labeling’ at a molecular level ensuring that the vesicle is delivered to a lysosome and not to the plasma membrane or elsewhere. The ‘label’ is returned to the Golgi apparatus for re-use (http://bscb.org/ Society for Cell Biology.org). This suggests that BORM primarily affects cell metabolism by the disruption of lysosomal function and thus initiating cell death. This view is supported by the changes in the apoptotic signaling cascades, i.e. the upregulation of Bcl-2 expression and further confirmed by a nearly complete reduction of mitochondrial O_2_ consumption (Fig. 8). Although the treatment with VSM also resulted in a significant reduction in respiration rate, the underlying mechanisms are different. Because of the stabilization of the actin cytoskeleton, the MG-63 cells are limited in their motility and can no longer divide, so that a G2/M arrest is forced.

## Conclusions

In this study two Pakistani plant extracts, namely VSM and BORM could be identified as potential anti-tumor agents at least on the bone and breast cancer cell lines in vitro. The mechanism of action of VSM is achieved by a cell cycle arrest in the G2/M phase and the stabilization of the cell by increased actin stress fiber formation. The antitumor effect of BORM is mediated by activating the lysosomal induced cell death pathway. However, both plant extracts exhibit strong cytotoxic potential in a concentration dependent manner. In this case VSM displayed the least impact on primary osteoblast functioning as non-tumorigenic cells whereas BORM showed the lowest cytotoxic effect on the mammary control cell line. Therefore, based on these results, we can postulate that VSM can be of interest for the treatment of bone tumors and BORM for the treatment of breast cancer. To prove this assertion, future work is on the identification of potential antitumor ingredients of these extracts and the evaluation of the dose-response relationships, in vitro and in vivo.

## Abbreviations

ANOVA, one-way analysis of variance; Bcl-2/BCL-2, B-cell lymphoma 2; BORM, *Berberis orthobotrys* roots; BOFM, *Berberis orthobotrys* fruits; BO-5, ethylacetate soluble oily substance of *Berberis orthobotrys* fruits; BO-23, n-hexane soluble oily substance of *Berberis orthobotrys* fruits; CMM, *Caccinia macranthera* aerial part; DAPI, 4’,6-diamidino-2-phenylindole; DMSO, dimethyl sulfoxide; DNA, Deoxyribonucleic acid; EtOH, ethanol; IC_50_, inhibitory concentration of 50 % population; MeOH, methanol; MTS, 3-(4, 5-dimethylthiazol-2-yl)-5-(3-carboxymethoxyphenyl)-2-(4-sulfophenyl)-2H-tetrazolium; OHRM, *Onosma hispida* roots; OHAM, *Onosma hispida* aerial parts; PCNA, Proliferating cell nuclear antigen; PI, propidium iodide; POB, primary osteoblast cells; SEM, standard error of mean or scanning electron microscopy; TCM, Traditional Chinese medicine; VSM, *Vincetoxicum arnottianum*

## Additional files

Additional file 1: Figure S1. Control cell cycle analysis. Histogram of cell cycle phases in MG-63 cells after treatment with 0.1 % of the control substances (DMSO, MeOH, EtOH) in comparison with untreated cells, cultivated in assay medium. No significant effect on the cell cycle phases, mediated by the solvents used in this study could be observed. (TIF 645 kb) Additional file 2: Figure S2. Bright field imaging of cell morphology. Bright field images of MG-63 cells after exposure to VSM or BORM (concentration series ranging from 1 to 100 μg/ml) for 48 h in comparison with the control treatment. (TIF 12518 kb) Additional file 3: Figure S3. Imaging of actin cytoskeleton. Laser scanning microscopic images of F-actin (red) and Hoechst (blue) stained MG-63 cells after exposure to VSM or BORM (concentration series ranging from 1 to 100 μg/ml) for 48 h in comparison with the control treatment. (TIF 12140 kb) Additional file 4: Figure S4. Live cell imaging of lysosomes. Laser scanning microscopic images of lysosomes (green) in MG-63 cells after treatment with 1–100 μg/ml BORM. Lysosomes were labeled with LysoTracker® Green DND-26 (Molecular Probes, Carlsbad, CA, USA). Cell nuclei (blue) were labeled with Hoechst. Notably, lysosome amount increased with rising BORM concentration. (TIF 6162 kb) Additional file 5: Figure S5. Metabolic live cell monitoring. Live cell monitoring of three metabolic parameters (extracellular acidification, mitochondrial O_2_ consumption and cell impedance) in MG-63 cells and primary osteoblasts (POB) during exposure to 25 μg/ml VSM or BORM in comparison to the control (which was set to 100 %) determined by the Bionas® 2500 analyzing system combined with the metabolic chip Bionas DisocveryTM SC1000. (TIF 1735 kb) Additional file 6: Figure S6. Wound healing assay. Raw data of the wound healing assay of VSM (25, 50 μg/ml) treated MG-63 cells. (TIF 10288 kb) Additional file 7: Figure S7. Apoptosis detection. Annexin V/PI labeling of VSM and BORM (50, 100 μg/ml) treated MG-63 cells. (TIF 1742 kb)
