# Supplementary figures and images for: Antitumor evaluation of two selected Pakistani plant extracts on human bone and breast cancer cell lines
Source: BMC Complement Altern Med. 2016 Jul 26;16:244. doi: 10.1186/s12906-016-1215-9 (PMC4960692; doi:10.1186/s12906-016-1215-9)

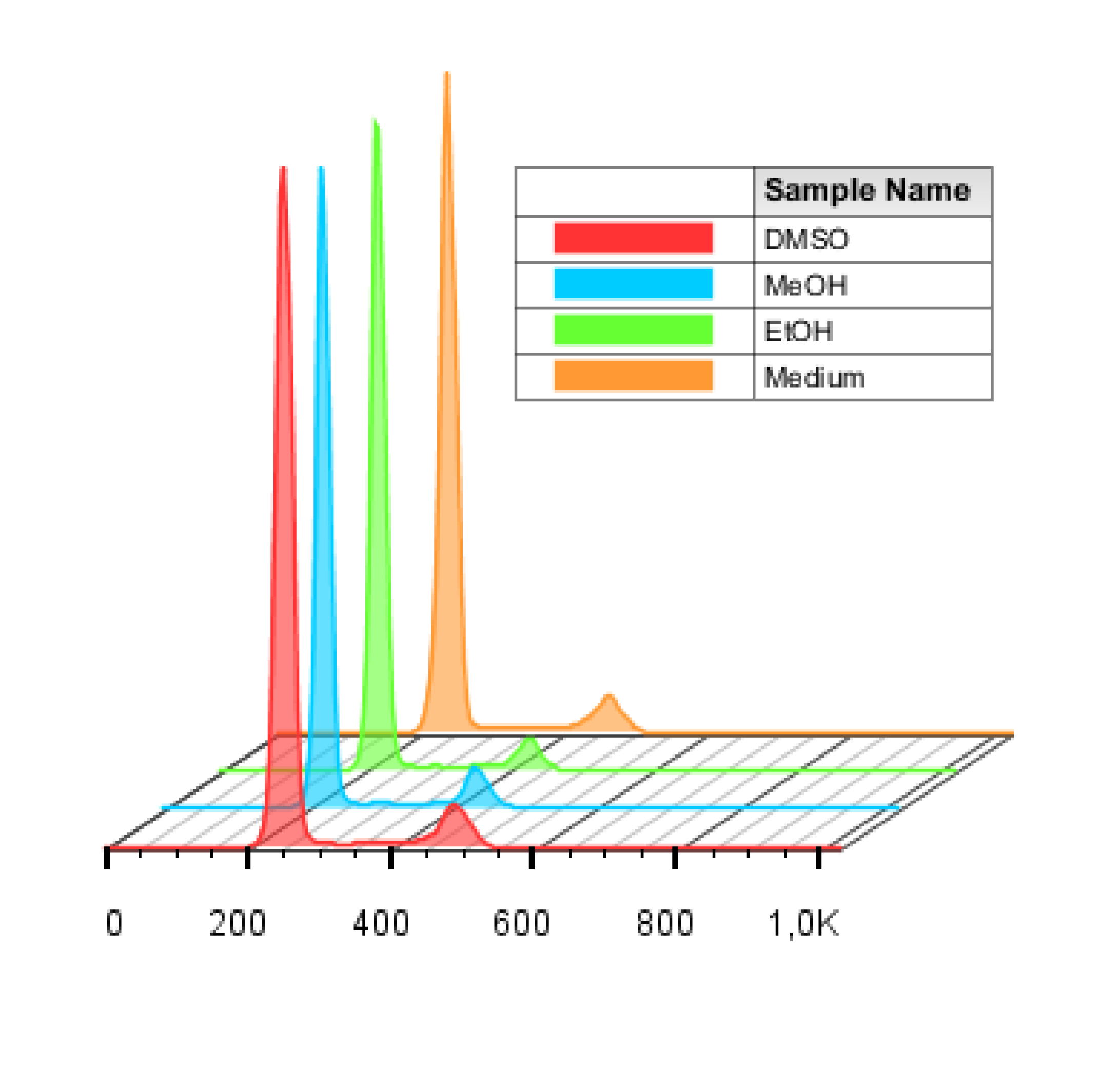

Supplement: Additional file 1: Figure S1. — Control cell cycle analysis. Histogram of cell cycle phases in MG-63 cells after treatment with 0.1 % of the control substances (DMSO, MeOH, EtOH) in comparison with untreated cells, cultivated in assay medium. No significant effect on the cell cycle phases, mediated by the solvents used in this study could be observed. (TIF 645 kb) [file 12906_2016_1215_MOESM1_ESM.tif]

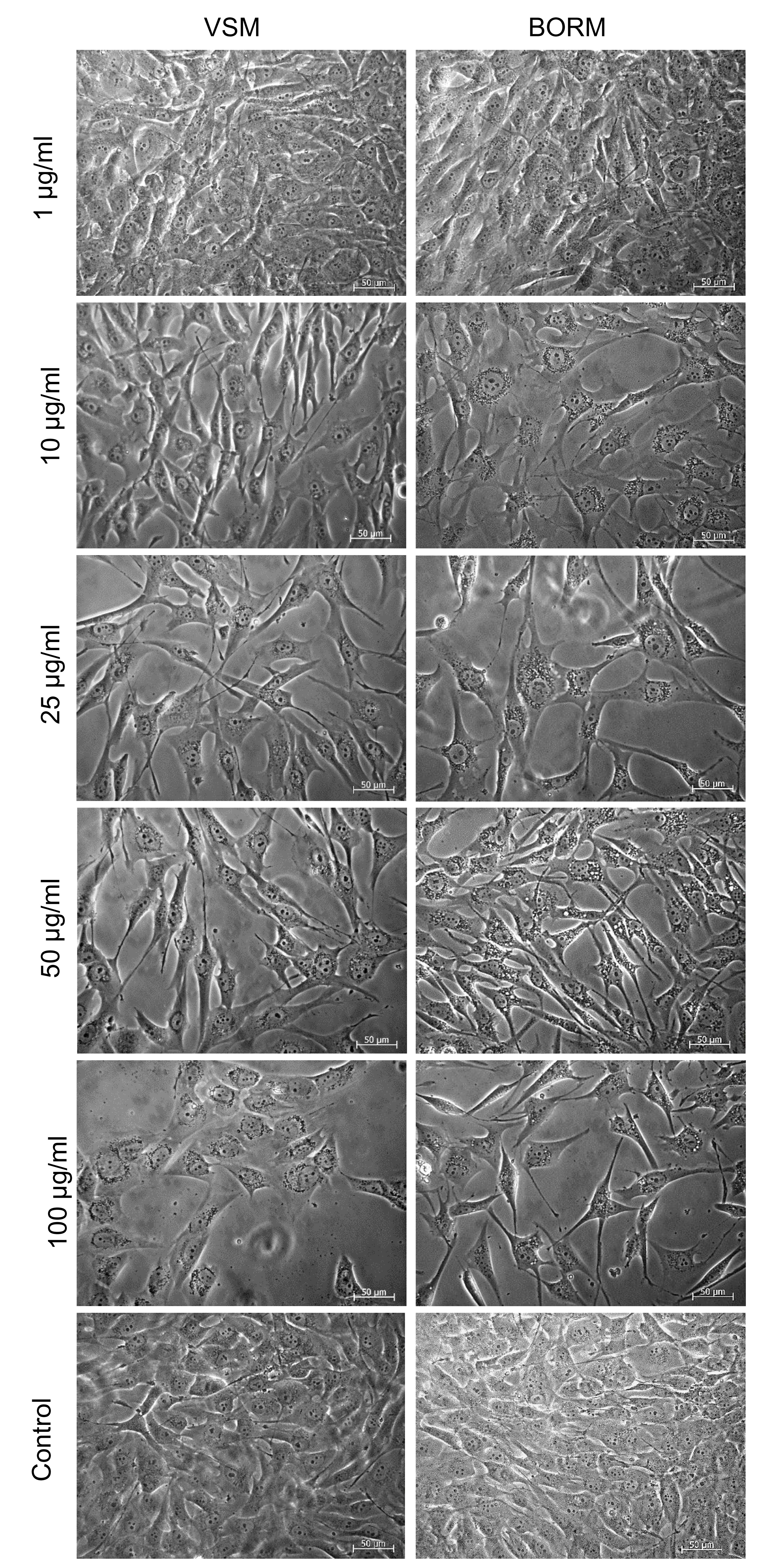

Supplement: Additional file 2: Figure S2. — Bright field imaging of cell morphology. Bright field images of MG-63 cells after exposure to VSM or BORM (concentration series ranging from 1 to 100 μg/ml) for 48 h in comparison with the control treatment. (TIF 12518 kb) [file 12906_2016_1215_MOESM2_ESM.tif]

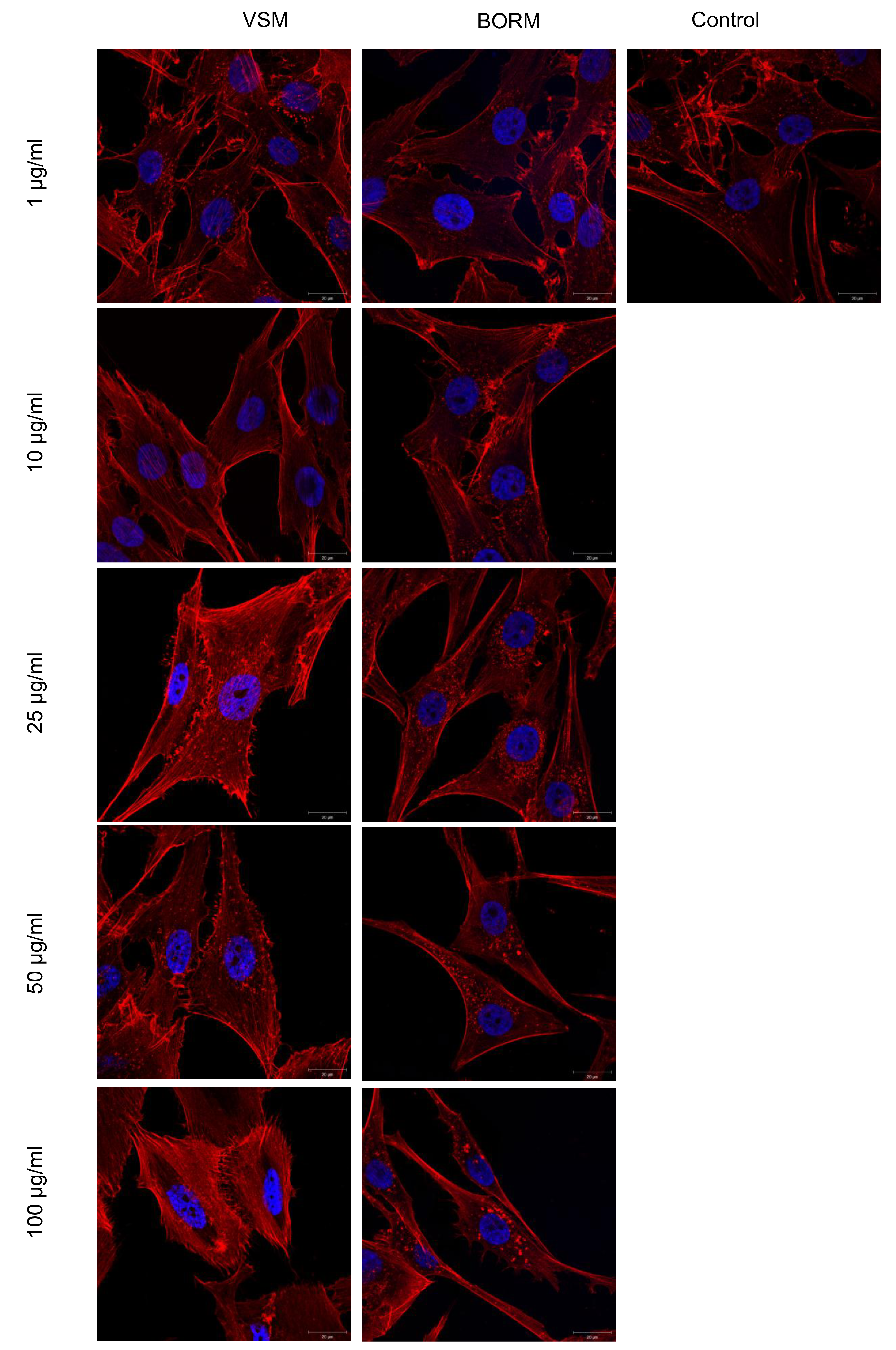

Supplement: Additional file 3: Figure S3. — Imaging of actin cytoskeleton. Laser scanning microscopic images of F-actin (red) and Hoechst (blue) stained MG-63 cells after exposure to VSM or BORM (concentration series ranging from 1 to 100 μg/ml) for 48 h in comparison with the control treatment. (TIF 12140 kb) [file 12906_2016_1215_MOESM3_ESM.tif]

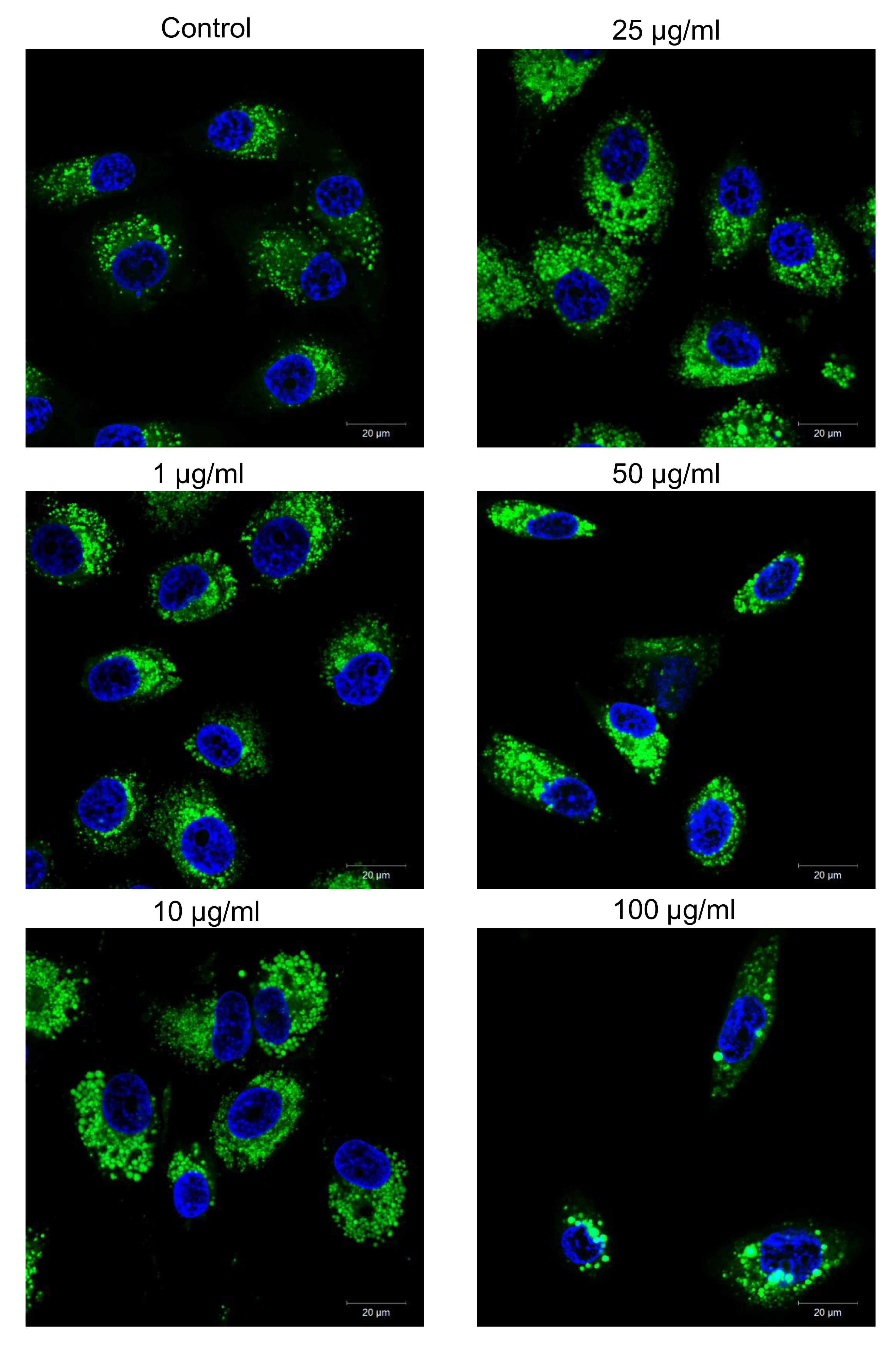

Supplement: Additional file 4: Figure S4. — Live cell imaging of lysosomes. Laser scanning microscopic images of lysosomes (green) in MG-63 cells after treatment with 1–100 μg/ml BORM. Lysosomes were labeled with LysoTracker® Green DND-26 (Molecular Probes, Carlsbad, CA, USA). Cell nuclei (blue) were labeled with Hoechst. Notably, lysosome amount increased with rising BORM concentration. (TIF 6162 kb) [file 12906_2016_1215_MOESM4_ESM.tif]

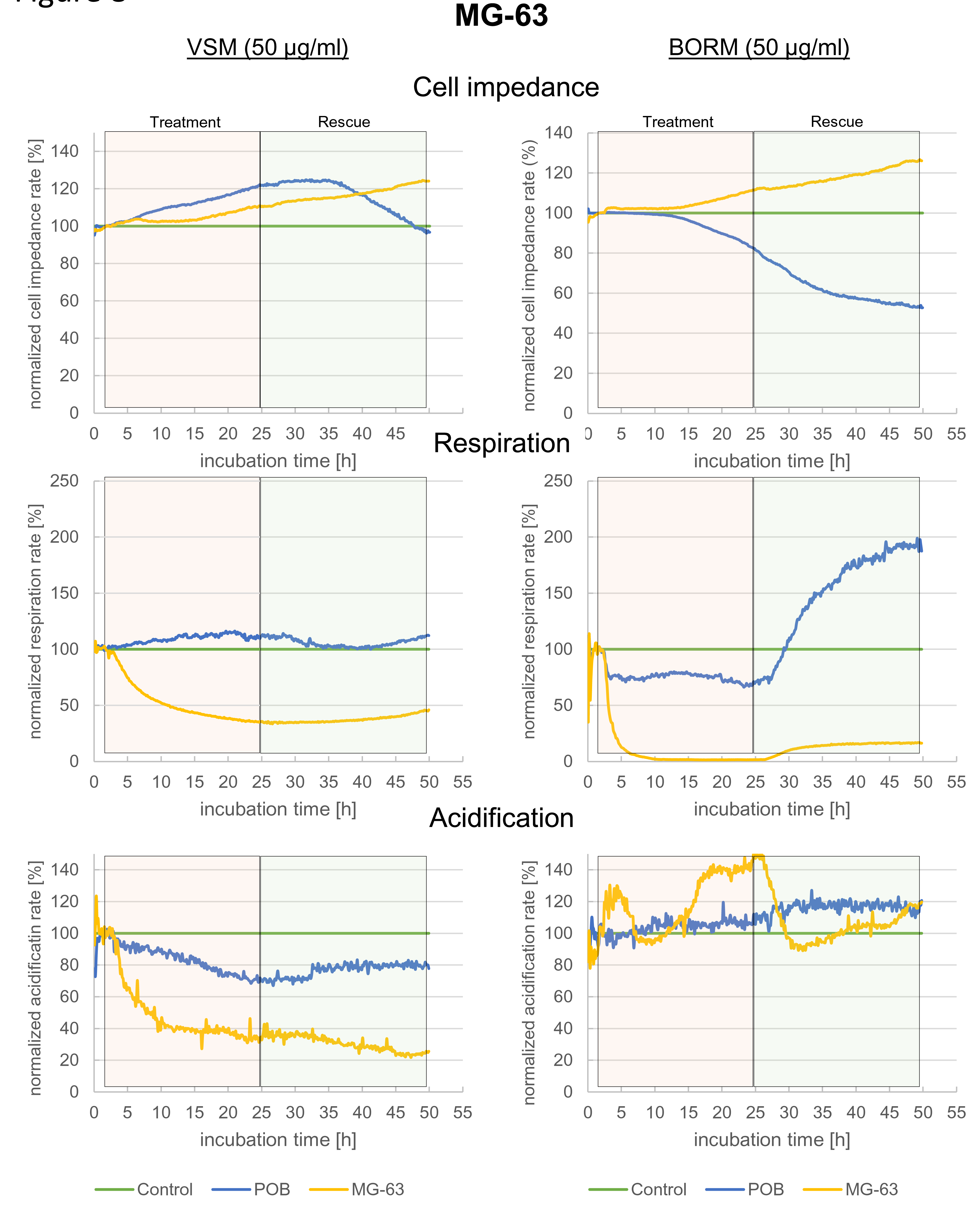

Supplement: Additional file 5: Figure S5. — Metabolic live cell monitoring. Live cell monitoring of three metabolic parameters (extracellular acidification, mitochondrial O2 consumption and cell impedance) in MG-63 cells and primary osteoblasts (POB) during exposure to 25 μg/ml VSM or BORM in comparison to the control (which was set to 100 %) determined by the Bionas® 2500 analyzing system combined with the metabolic chip Bionas DisocveryTM SC1000. (TIF 1735 kb) [file 12906_2016_1215_MOESM5_ESM.tif]

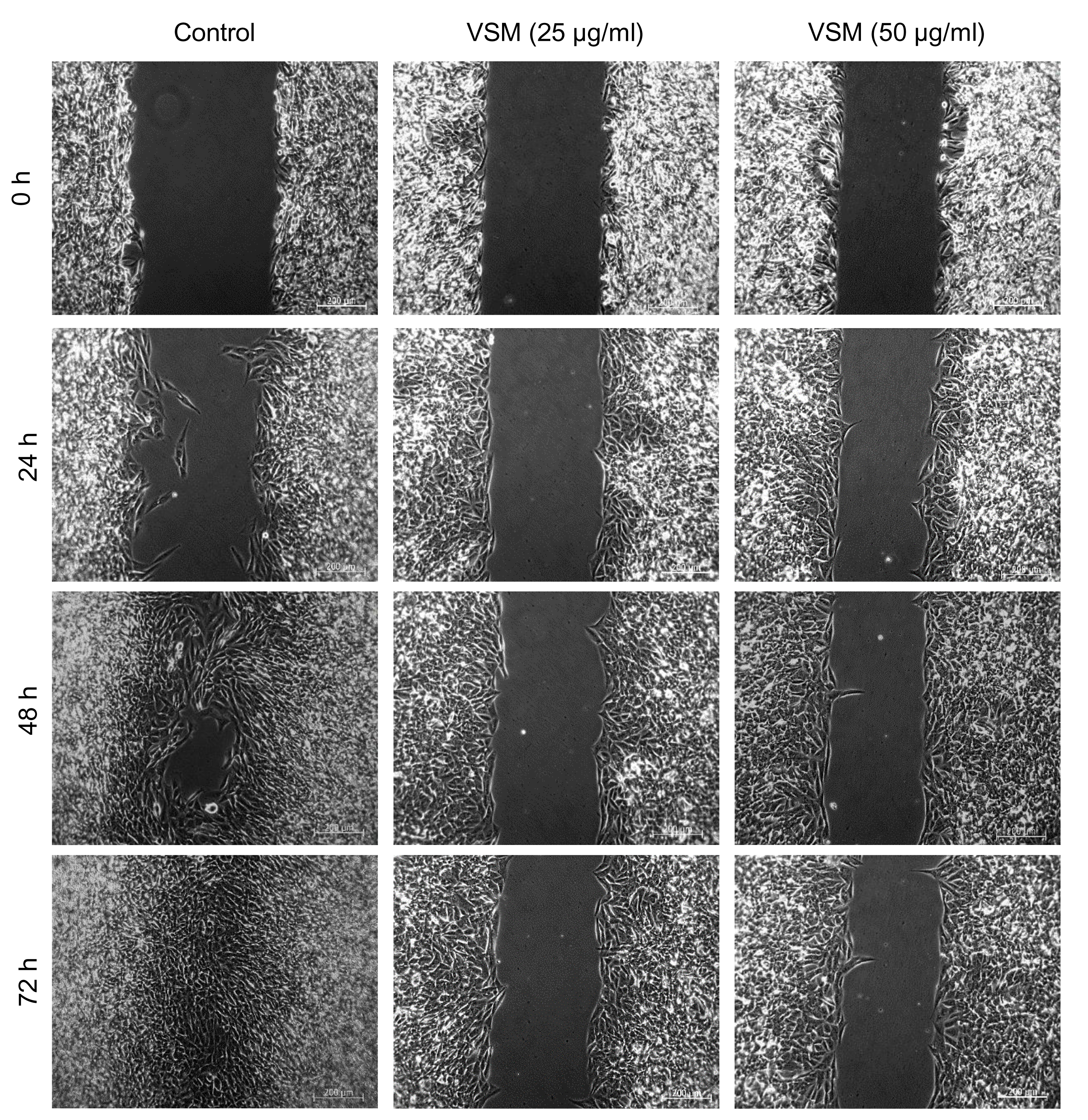

Supplement: Additional file 6: Figure S6. — Wound healing assay. Raw data of the wound healing assay of VSM (25, 50 μg/ml) treated MG-63 cells. (TIF 10288 kb) [file 12906_2016_1215_MOESM6_ESM.tif]

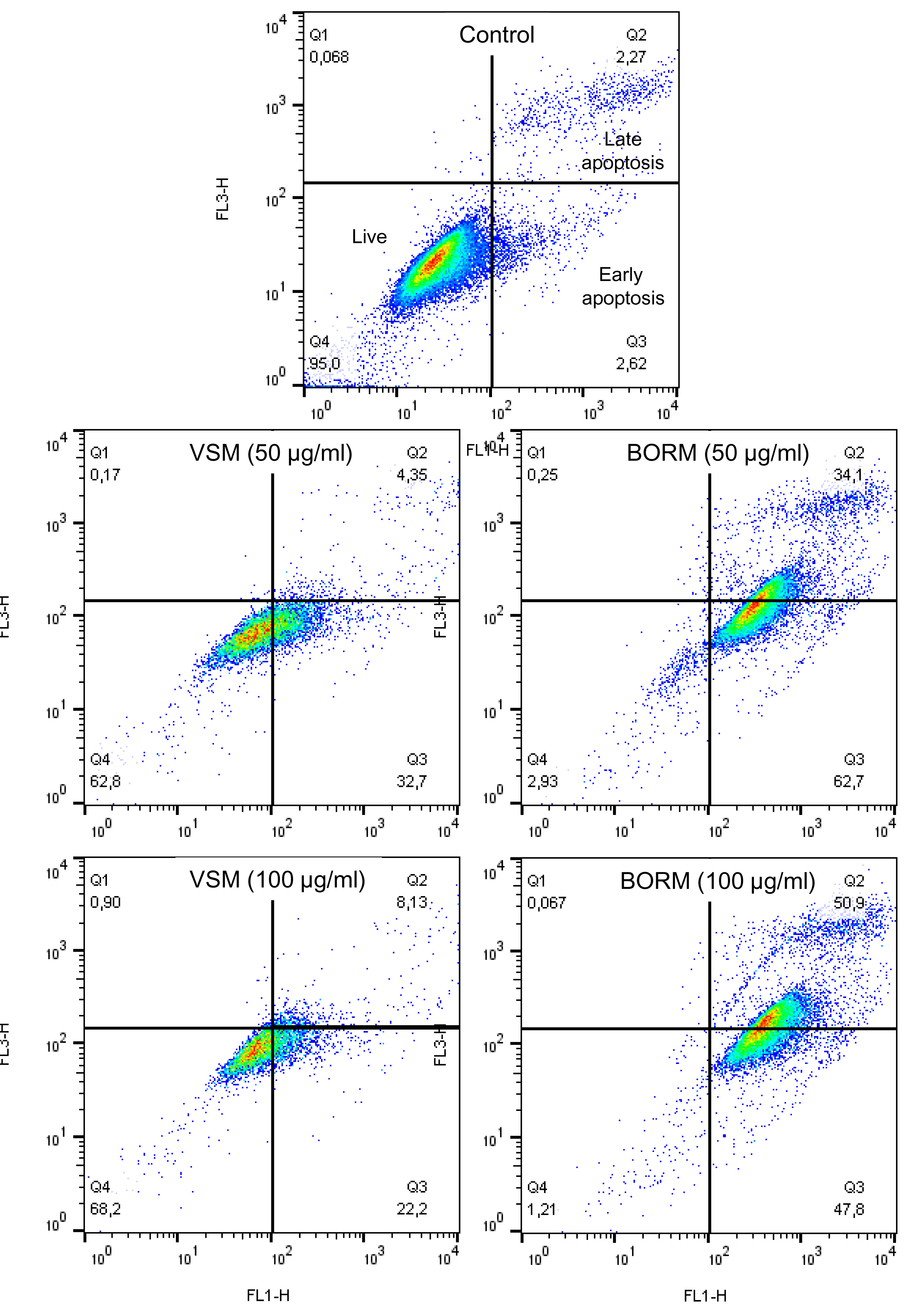

Supplement: Additional file 7: Figure S7. — Apoptosis detection. Annexin V/PI labeling of VSM and BORM (50, 100 μg/ml) treated MG-63 cells. (TIF 1742 kb) [file 12906_2016_1215_MOESM7_ESM.tif]
